# Molecular characterization of *Salmonella* isolates from poultry in Egypt: Detection of virulence, ESBLs genes and antimicrobial resistance profiling

**DOI:** 10.1007/s11259-025-10936-0

**Published:** 2025-11-18

**Authors:** Mona Salem, Reem Ghabbour, Noura F. Mostafa, Amal Awad, Gamal Younis

**Affiliations:** 1 Department of Bacteriology, Mycology and Immunology, Faculty of Veterinary Medicine, Mansoura University, Mansoura, 35516 Egypt; 2 Gastro- Enterology Surgery Center (GEC), Mansoura University, Mansoura, 35516 Egypt

**Keywords:** Salmonella, Broiler chicken, Serotyping, Virulence, Antimicrobial susceptibility, MDR, XDR, ESBLs

## Abstract

This study aimed to determine the prevalence, serovars, virulence gene profiles, antimicrobial resistance patterns, and extended-spectrum β-lactamase (ESBL) genes in *Salmonella* isolated from broiler chickens in Egypt. In this study, 200 samples were obtained from broiler chickens. Samples were processed using standard bacteriological techniques, and suspected isolates were biochemically identified and molecularly confirmed by PCR targeting the *inv*A gene. Serotyping was performed according to the Kauffmann–White scheme. Virulence genes (*hil*A, *stn*, *spv*C, and *spi*C) and ESBL genes (*bla*_TEM_, *bla*_SHV_, and *bla*_*CTX*−M_) were detected by PCR. Antimicrobial susceptibility testing was carried out following CLSI guidelines. Nineteen isolates (9.5%) were confirmed as *Salmonella*. Serotyping revealed four serovars: *S*. Kentucky (52.63%), *S*. Typhimurium (21.05%), *S*. Salamae (15.79%), and *S*. Infantis (10.53%). All isolates carried *inv*A and *hil*A, while *stn* and *spi*C were present in 84.21% and 73.68% of isolates, respectively, *spv*C was detected in only one isolate (5.26%). All isolates were resistant to ceftazidime, cefepime, and erythromycin. High resistance rates were also observed against amoxicillin, amoxicillin-clavulanic acid, ciprofloxacin, and doxycycline. Detection of ESBL genes revealed that *bla*_TEM_ was present in 16 isolates (84.2%), *bla*_CTX−M_ in 6 isolates (31.6%), and *bla*_SHV_ in 2 isolates (10.5%). Statistical analyses (Chi-square and Spearman’s correlation tests) further confirmed significant associations between ESBL genes and both phenotypic resistance and virulence determinants, strengthening the validity of our findings. The high prevalence of virulence factors and antimicrobial resistance traits, particularly extensively drug-resistant (XDR) phenotypes and ESBL-encoding genes among *Salmonella* isolates from poultry, represents a critical public health concern. These findings emphasize the need for continuous molecular surveillance, improved antimicrobial stewardship, and enhanced biosecurity strategies in poultry production in Egypt.

**Supplementary Information:**

The online version contains supplementary material available at 10.1007/s11259-025-10936-0.

## Introduction

*Salmonella* is one of the most significant foodborne pathogens worldwide and is commonly associated with poultry products. Chickens serve as reservoirs for *Salmonella* and can transmit it to humans, leading to gastrointestinal infections ranging from mild to severe. In some cases, the infection can become systemic. *Salmonella* infection in chickens can occur either vertically from hen to egg or horizontally via contaminated feed, water, litter, or handlers (Shaji et al. 2023).

The severity of *Salmonella* infection in chickens varies depending on the serovar and the age of the birds. Acute infections may cause symptoms such as depression, ruffled feathers, decreased feed intake, diarrhea, dehydration, stunted growth, huddling, and increased mortality, particularly among young chicks. Systemic infections caused by *S*. Pullorum or *S*. Gallinarum may cause signs such as a white, pasty vent, labored breathing, swollen joints, and hepatosplenomegaly upon postmortem examination. However, many infections, particularly those caused by non-host-specific serovars, remain subclinical, which makes detection and control more challenging (Markos 2017).

Once inside a host, *Salmonella* activates various virulence determinants to survive and multiply while evading the immune system. These genes are often encoded on *Salmonella* pathogenicity islands (SPIs), plasmids, or other mobile genetic elements and are tightly regulated. One of these genes is the *inv*A gene, which is considered one of the most conserved and widely used molecular markers for identifying *Salmonella* species. The *inv*A gene encodes a component of the Type III Secretion System (T3SS-1), which plays a critical role in invading host intestinal epithelial cells. The *inv*A gene is located on *Salmonella* pathogenicity island 1 (SPI-1) and is essential for bacterial entry into non-phagocytic cells. This makes it a key virulence factor in the pathogenesis of salmonellosis (Mashayekh et al. 2022).

Furthermore, HilA is a transcriptional regulator protein located within SPI-1 that activates the expression of invasion genes. This enables *Salmonella* to penetrate intestinal epithelial cells, which is the first step in human gastroenteritis (Han et al. 2024). *Stn* (*Salmonella* enterotoxin gene): It encodes an enterotoxin that is responsible for fluid accumulation in the intestine and contributes to diarrhea. It is considered a marker of enteric virulence (Nakano et al. 2012). Additionally, *Spv*C (*Salmonella* plasmid virulence gene): Located on the *Salmonella* virulence plasmid, this gene contributes to systemic infection by interfering with host cell signaling and promoting survival and replication in macrophages within human immune cells, leading to extraintestinal infections such as sepsis, especially in immunocompromised individuals. *Spi*C is encoded on SPI-2 and plays a central role in inhibiting phagosome-lysosome fusion, which allows *Salmonella* to survive and replicate (Han et al. 2024).

In recent years, the emergence of antibiotic-resistant *Salmonella* strains has become a major public health concern. The overuse and misuse of antimicrobials in poultry production for therapeutic and growth-promoting purposes exerts selective pressure that leads to the development and spread of resistant strains. Infections caused by MDR strains are associated with higher morbidity, longer hospital stays, and increased healthcare costs (Abreu et al. 2023).

Extended-spectrum β-lactamases (ESBLs), particularly TEM, SHV, and CTX-M, represent a major mechanism of resistance against β-lactam antibiotics among Gram-negative pathogens such as *Salmonella enterica*. These enzymes hydrolyze penicillins, cephalosporins, and aztreonam, rendering treatment options limited and leading to therapeutic failure in both human and veterinary medicine (Rawat and Nair 2010; Dandachi et al. 2018). Among these, CTX-M-type enzymes have emerged as the most prevalent globally, surpassing the older TEM and SHV variants in both clinical and environmental settings (Bevan et al. 2017). From a veterinary perspective, ESBL-producing *Salmonella* strains can lead to recurrent infections, reduced treatment efficacy, and increased mortality in poultry flocks, ultimately causing economic losses (EFSA 2021). In humans, infections caused by ESBL-producing *Salmonella* are associated with longer hospital stays, delayed effective therapy, and increased risk of complications, particularly in vulnerable populations such as children, the elderly, and immunocompromised individuals (Crump et al. 2015).

In Egypt, several recent studies have highlighted the persistence and dissemination of multidrug-resistant (MDR) and extensively drug-resistant (XDR) *Salmonella* strains in poultry production systems. For instance, migratory birds were identified as a potential source of resistant *Salmonella enterica* transmission to broiler farms and their environment (Tawakol et al. 2024). Similarly, Elsayed et al. (2024) reported a high ecological prevalence and genetic diversity of *Salmonella* Enteritidis with marked multidrug resistance in broiler and layer chicken farms. In another investigation, Algammal et al. (2023) characterized XDR *S*. Enteritidis and *S*. Typhimurium, showing strong associations with virulence and resistance genes. Moreover, Orabi et al. (2022) and Elhariri et al. (2020) documented the zoonotic risk of ESBL-producing *Salmonella* strains in poultry, poultry workers, and humans, underlining the One Health concern in the Egyptian context. Therefore, simultaneous monitoring of virulence factors and antimicrobial resistance in *Salmonella* from poultry is crucial to assess their zoonotic potential, guide prudent antibiotic use, and inform policy-making in the fields of food safety and public health (Beceiro et al. 2013). This study aimed to determine the prevalence, serovars, virulence gene profiles (*inv*A, *hil*A, *stn*, *spv*C, and *spi*C), and antimicrobial resistance patterns of *Salmonella* spp. isolated from broiler chicken samples, in addition to the molecular detection of extended-spectrum β-lactamase (ESBL) genes (*bla*_TEM_, *bla*_SHV_, and *bla*_CTX-M_) to protect public health and enhance surveillance efforts.

## Materials and methods

### Ethical approval

Ethical clearance for the study was granted by the Research Ethics Committee of the Faculty of Veterinary Medicine, Mansoura University, Egypt (Protocol code: MU-ACUC (VM.PhD.23.10.24). All procedures involving poultry were carried out in strict accordance with international ethical standards, ensuring the humane treatment of animals and the protection of farm personnel throughout the study.

### Sample collection

A total of 200 chicken samples were collected over the period from March to May 2024 from broiler chickens aged between 1 and 40 days across 17 private chicken farms (10 samples from each) and 3 diagnostic veterinary laboratories (10 samples from each) serving chicken farms in Dakahlia Governorate, including Mansoura City and neighboring rural areas. The samples included cloacal swabs, liver, spleen, heart, unabsorbed yolk sacs, and intestinal contents, collected aseptically from clinically ill and freshly slaughtered birds suspected to be infected with *Salmonella*. All samples were collected under sterile conditions and transported in cold boxes maintained at 4 °C. Samples were then immediately transferred to the Department of Bacteriology, Mycology, and Immunology, Faculty of Veterinary Medicine, Mansoura University, Egypt, and were processed within 4–6 h of collection to ensure bacterial viability and minimize contamination.

According to farm records, management practices and antimicrobial use indicated that the birds were conventionally raised, and antimicrobial agents were administered during the rearing cycle. During the first 10 days (starter period), the most frequently used antimicrobials included florfenicol, colistin, thiamphenicol, and doxycycline. At later stages, antimicrobials such as fosfomycin, levofloxacin, difloxacin, apramycin, flumequine, and amoxicillin were occasionally used for the treatment of enteric infections.

### Isolation and identification of Salmonella spp

Pooled visceral organs (spleen, heart, liver, and unabsorbed yolk sacs), along with cloacal swabs, were pre-enriched in Buffered Peptone Water (BPW) (Oxoid Ltd, England) and incubated at 37 °C for 18 h. Then, 0.1 mL of the pre-enriched culture was transferred to Rappaport-Vassiliadis (RV) (Oxoid Ltd, England) broth and incubated at 42 °C for 24 h for selective enrichment. Intestinal samples were handled independently to avoid any risk of cross-contamination while adhering to the same pre-enrichment and incubation procedures. A loopful from the RV broth was streaked onto Xylose Lysine Deoxycholate (XLD) agar and MacConkey’s agar plates (Oxoid Ltd, England), followed by incubation at 37 °C for 24 h. Typical *Salmonella* colonies (red with black centers on XLD or pale, non-lactose-fermenting on MacConkey’s agar) were picked and subjected to Gram staining and biochemical identification using Triple Sugar Iron (TSI), Methyl Red (MR), Urease test, Indole test, and Citrate utilization. Confirmed isolates were stored at − 20 °C in Tryptic Soy Broth (TSB) with 20% glycerol for further molecular analysis (ISO 6579:2002 standards).

### Genotypic confirmation of Salmonella spp. By PCR

Genomic DNA was extracted from retrieved isolates using the boiling method. A loopful of overnight culture was suspended in 200 µL of sterile distilled water, boiled in a thermal block (Biometra) at 100 °C for 10 min, and then centrifuged at 12,000 rpm for 5 min. The supernatant was used as a DNA template (Salem et al. 2023). For molecular confirmation of *Salmonella* isolates, the polymerase chain reaction (PCR) assay targeting the *inv*A gene was performed. The PCR was carried out according to the protocol of Nadi et al. (2020). The sequence of the primer and expected amplicon size are listed in Table 1. PCR amplification was performed in a total reaction volume of 25 µL, comprising 12.5 µL of DreamTaq Green PCR Master Mix (Thermo Scientific, USA), 1 µL of each primer (10 pmol), 5.5 µL of nuclease-free water, and 5 µL of extracted DNA template. The PCR reactions were performed using a 2720 Thermal Cycler (Applied Biosystems, Thermo Fisher, USA). Thermal cycling was conducted under the following conditions: initial denaturation at 94 °C for 2 min; 35 cycles of denaturation at 95 °C for 1 min, annealing at 62 °C for 30 s, and extension at 72 °C for 30 s; followed by a final extension at 72 °C for 10 min. PCR products were resolved by gel electrophoresis on a 1% agarose gel prepared in 1X TBE buffer, stained with ethidium bromide, and electrophoresed at 100 V for 45 min. A 1000 bp DNA ladder (Qiagen, USA) served as the molecular weight marker. DNA bands were visualized under UV illumination using a Cleaver Scientific transilluminator system (UK). To ensure amplification specificity, a negative control (nuclease-free water) was included in each PCR run.

**Table 1:** Oligonucleotide primers used in this study

Target gene	Primer direction and sequence	Amplicon size (bp)		Reference
*inv*A	F: GTGAAATTATCGCCACGTTCGGGCAA	284	Nadi et al. (2020)	
	R: TCATCGCACCGTCAA AGGAACC			
*spvC*	F: ACTCCTTGCACAACCAAATGCGGA	571	Krzyzanowski et al. (2014)	
	R: TGTCTTCTGCATTTCGCCACCATCA			
*stn*	F: CTTAATCGCGCCGCCATGCTGTT	480	El-Saeed et al. (2024)	
	R: CATGAACTGGCGCAGGTGAT			
*hil*A	F: CGTGAAGGGATTATCGCAGT	296	Kong-Ngoen et al. (2022)	
	R: GTCCGGGAATACATCTGAGC			
*spi*C	F: CCTGGATAATGACTATTGAT	309	Odjadjare and Olaniran (2015)	
	R: AGTTTATGGTGATTGCGTAT			
*bla*_TEM_	F: ATCAGCAATAAACCAGC	516	Colom et al. (2003)	
	R: CCCCGAAGAACGTTTTC			
*bla*_SHV_	F: AGGATTGACTGCCTTTTTG	392	Colom et al. (2003)	
	R: ATTTGCTGATTTCGCTCG			
*bla*_CTX−M_	F: ATGTGCAGYACCAGTAARGTKATG GC	593	Archambault et al. (2006)	
	R: TGGGTRAARTARGTSACCAGAAYCAGC GG			

*F* Forward primer, *R* Reverse primer, *bp* base pair

### Serological identification of Salmonella isolates

*Salmonella* isolates were subsequently subjected to serotyping to identify their somatic (O) and flagellar (H) antigens. The slide agglutination technique was employed using commercially available antisera (SISIN, Berlin). Serotyping was conducted according to ISO 6579:2014, based on the Kauffmann–White classification scheme. The procedure was carried out at the National *Salmonella* Reference Laboratory at the Animal Health Research Institute (AHRI), Dokki, Egypt, which is an ISO/IEC 17,025 accredited facility by the Egyptian Accreditation Council (EGAC).

### Molecular detection of virulence genes by PCR

Specific virulence genes, including *spv*C, *stn*, *hil*A, and *spi*C, were amplified using gene-specific primers (Table 1). A PCR program included: Initial denaturation: 95 °C for 5 min, 30 cycles of: Denaturation: 94 °C for 30 s, annealing at 58 °C for 30 s, extension: 72 °C for 45 s, and final extension: 72 °C for 10 min. PCR products were separated by agarose gel electrophoresis (1%), stained with ethidium bromide, and visualized under UV transillumination. A 1000 bp DNA ladder was used to estimate product size.

### Phenotypic evaluation of antimicrobial resistance in Salmonella isolates

Antimicrobial resistance in *Salmonella* isolates was examined using the disk diffusion approach on Mueller–Hinton agar (Oxoid Ltd., England), following the 2024 standards outlined by the Clinical and Laboratory Standards Institute (CLSI 2024) (Table 2). Antimicrobial discs (*n* = 13) (Oxoid Ltd, Hampshire, England) representing multiple antimicrobial classes (*n* = 9) of both human and veterinary importance were used. Tested antibiotics: (Aminopenicillin): Amoxicillin (AX; 25 µg), β-lactamase inhibitor: Amoxicillin-clavulanic acid (AMC; 30 µg), Cephalosporin: Ceftriaxone (CTR; 30 µg), Ceftazidime (CAZ; 30 µg), Cefepime (CPM; 30 µg), Carbapenems: Meropenem (MEM; 10 µg), Phenicol: Chloramphenicol (C; 30 µg), Tetracyclines: Doxycycline (DO; 30 µg), Fluoroquinolones: Ciprofloxacin (CIP; 5 µg), Macrolides: Erythromycin (E; 15 µg), Aminoglycoside: Gentamicin (CN; 10 µg), Streptomycin (S; 10 µg), DHFR inhibitor + Sulfonamides: Trimethoprim/sulfamethoxazole (SXT; 25 µg). Plates were incubated at 37 °C for 24 h, and the diameter of the inhibition zones was measured and interpreted according to CLSI breakpoints. Each isolate was interpreted as susceptible, intermediate, or resistant according to its inhibition zone diameter. Multidrug-resistant (MDR) *Salmonella* isolates were classified based on the guidelines proposed by Magiorakos et al. (2012), which define MDR as non-susceptibility to at least one antimicrobial agent in three or more distinct antimicrobial classes. Furthermore, isolates exhibiting resistance to all tested antibiotics except for one or two antimicrobial categories were categorized as extensively drug-resistant (XDR), while those showing resistance to all antimicrobial agents across all tested classes were defined as pandrug-resistant (PDR), as described by Rahman et al. (2024). The multidrug resistance index (MDRI) was quantified as the proportion of antibiotics resisted by each isolate relative to the total number of antimicrobial agents assessed (Rahman et al. 2024).

**Table 2:** Interpretation and classification of antimicrobial susceptibility for *Salmonella* serovars according to CLSI guidelines (2024)

Antibiotic	Disc Conc.	Class	Sub-class	Zone Diameter (mm) (*R* ≤/I/S ≥)
Amoxicillin (AX)	25 µg	β-lactams	Aminopenicillins	≤ 13/14–16/≥17
Amoxicillin-clavulanic acid (AMC)	30 µg	β-lactams	Penicillin + β-lactamase inhibitor	≤ 13/14–17/≥18
Ceftriaxone (CTR)	30 µg	β-lactams	Cephalosporin	≤ 19/20–22/≥23
Ceftazidime (CAZ)	30 µg	β-lactams	Cephalosporin	≤ 17/18–20/≥21
Cefepime (CPM)	30 µg	β-lactams	Cephalosporin	≤ 17/18–20/≥21
Meropenem (MEM)	10 µg	β-lactams	Carbapenems	≤ 14/15–17/≥18
Chloramphenicol (C)	30 µg	Phenicol	-	≤ 12/13–17/≥18
Doxycycline (DO)	30 µg	Tetracyclines	-	≤ 12/13–15/≥16
Ciprofloxacin (CIP)	5 µg	Fluoroquinolones	-	≤ 20/21–30/≥31
Erythromycin (E)	15 µg	Macrolides	-	≤ 13/14–22/≥23
Gentamicin (CN)	10 µg	Aminoglycoside	-	≤ 12/13–14/≥15
Streptomycin (S)	10 µg	Aminoglycoside	-	≤ 11/12–13/≥14
Trimethoprim/sulfamethoxazole (SXT)	25 µg	Folate pathway inhibitors	DHFR inhibitor + Sulfonamides	≤ 10/11–15/≥16

*AX* Amoxicillin, *AMC* Amoxicillin-clavulanic acid, *CTR* Ceftriaxone, *CAZ* Ceftazidime, *CPM* Cefepime, *MEM* Meropenem, *C* *Chloramphenicol* *DO* Doxycycline, *CIP* Ciprofloxacin, *E* Erythromycin *CN* Gentamicin, *S* *Streptomycin*, *SXT* Trimethoprim/sulfamethoxazole, *DHFR* Dihydrofolate reductase, *R* Resistant, *I* Intermediate, *S* Susceptible

### Genotypic detection of ESBL genes

The detection of ESBL-encoding genes (*bla*_TEM_, *bla*_SHV,_ and *bla*_CTX−M_) was carried out using conventional PCR with specific primers. The sequences of the primers and expected amplicon sizes are listed in Table 1. PCR amplification was carried out using the following thermal profile: an initial denaturation step at 94 °C for 5 min, followed by 35 cycles consisting of denaturation at 94 °C for 30 s, primer annealing at 57 °C for 30 s, and strand extension at 72 °C for 1 min, ending with a final extension phase at 72 °C for 7 min. Amplified PCR products were run on a 1% agarose gel stained with ethidium bromide, and bands were visualized under UV illumination.

### Statistical analysis

Antimicrobial susceptibility data were dichotomized into resistant and susceptible/intermediate categories according to the Clinical and Laboratory Standards Institute (CLSI) guidelines. The presence of ESBL-encoding genes (*inv*A, *hil*A, *stn*, *spv*C, *spi*C, *bla*_TEM_, *bla*_SHV_, and *bla*_CTX−M_) was recorded as binary variables (present = 1, absent = 0). Associations between the presence of each ESBL gene and phenotypic resistance to individual antimicrobial agents were evaluated using the Chi-square (χ²) test of independence. For contingency tables in which more than 20% of the expected cell counts were less than five, Fisher’s exact test was applied. The strength and significance of the associations were interpreted based on the χ² statistic and the corresponding p-value, where statistical significance was defined as *p* < 0.05 and high statistical significance as *p* < 0.01. In addition, associations between ESBL-encoding genes and virulence determinants (*inv*A, *hil*A, *stn*, *spv*C, and *spi*C) were assessed using Spearman’s rank correlation analysis. The correlation coefficient (ρ) was calculated to determine the direction and magnitude of the relationship, with p-values used to assess statistical significance. Legend for significance levels: *p* < 0.001 – Highly significant, *p* < 0.01 – Very significant, *p* < 0.05 – Significant. The correlation matrix was created in Python (version 3.10) utilizing the scipy, pandas, and seaborn packages, with visualization performed through seaborn version 0.11.2. Additional heatmaps for alternative datasets were produced using Microsoft Excel 2019 and TBtools version 1.098.

## Results

### Prevalence, isolation, and molecular identification of Salmonella isolates

**Fig. 1:**
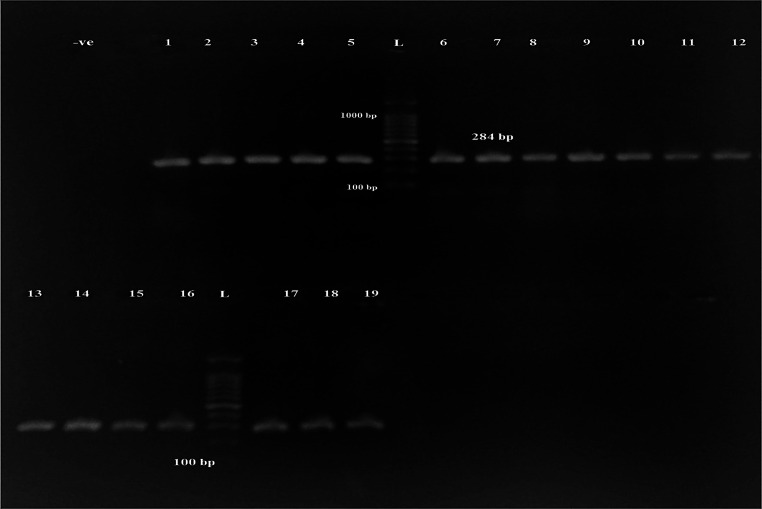
PCR amplification of the *inv*A gene (*n* = 19) at 284 bp, (L): 1000 bp DNA ladder; bp: base pairs

A total of 200 chicken samples were cultured on Xylose-Lysine-Desoxycholate (XLD) agar and MacConkey agar. Based on characteristic colony morphology, 30 isolates (15%) were initially suspected to be *Salmonella*, as they appeared as red colonies with black centers on XLD agar and as pale, non-lactose-fermenting colonies on MacConkey agar. Gram staining of the suspected colonies revealed Gram-negative, rod-shaped bacilli. Thirty presumptive *Salmonella* isolates were subjected to biochemical testing. Based on biochemical profiles, 19 isolates (63.3%) were confirmed as *Salmonella*. Molecular identification was then performed by PCR amplification of the *inv*A gene, which confirmed that all 19 biochemically identified isolates (100%) harbored the *inv*A gene (Fig. 1), corresponding to an overall prevalence of 9.5% (19/200). Among confirmed isolates, 6 (31.6%) were obtained from cloacal swabs, while 13 (68.4%) were recovered from internal organ samples.

### Serological identification of Salmonella isolates

**Fig. 2:**
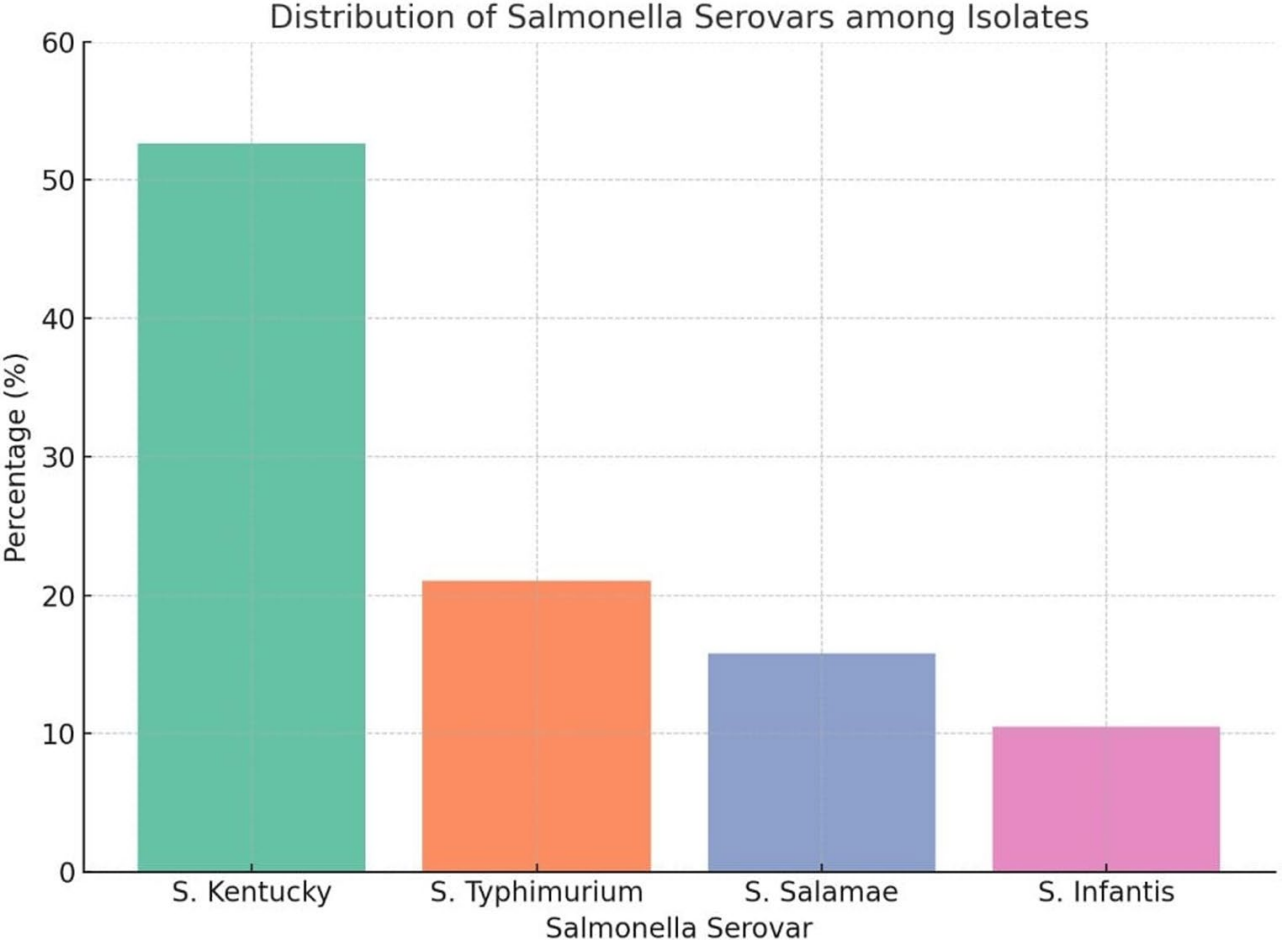
Distribution of *Salmonella* serovars among the isolates (*n* = 19). The most frequently identified serovar was *S*. Kentucky (52.63%), followed by *S*. Typhimurium (21.05%), *S*. Salamae (15.79%), and *S*. Infantis (10.53%). Each bar represents the percentage of isolates for each serovar

The *Salmonella* isolates underwent serological typing to determine their somatic (O) and flagellar (H) antigens. The serotyping results demonstrated the presence of four distinct *Salmonella* serovars among the confirmed isolates. The most frequently identified serovar was *S.* Kentucky, detected in 10 out of 19 isolates (52.63%), followed by *S*. Typhimurium in 4 isolates (21.05%), *S.* Salamae in 3 isolates (15.79%), and *S*. Infantis in 2 isolates (10.53%) (Fig. 2).

### Detection of virulence genes by PCR

**Fig. 3:**
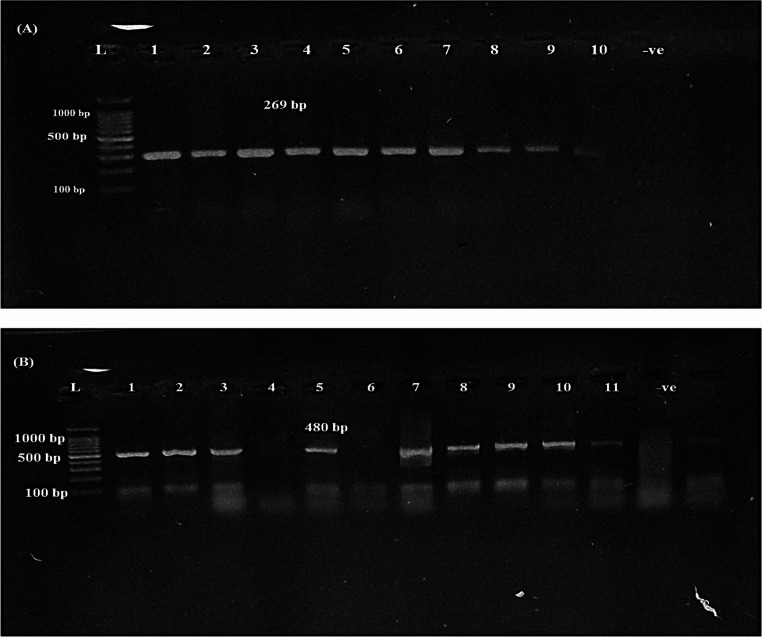
PCR amplification of virulence genes in *Salmonella* isolates. (**A**) *hil*A gene at 269 bp, (**B**) *stn* gene at 480 bp. (L) 1000 bp DNA ladder; bp: base pairs

**Fig. 4:**
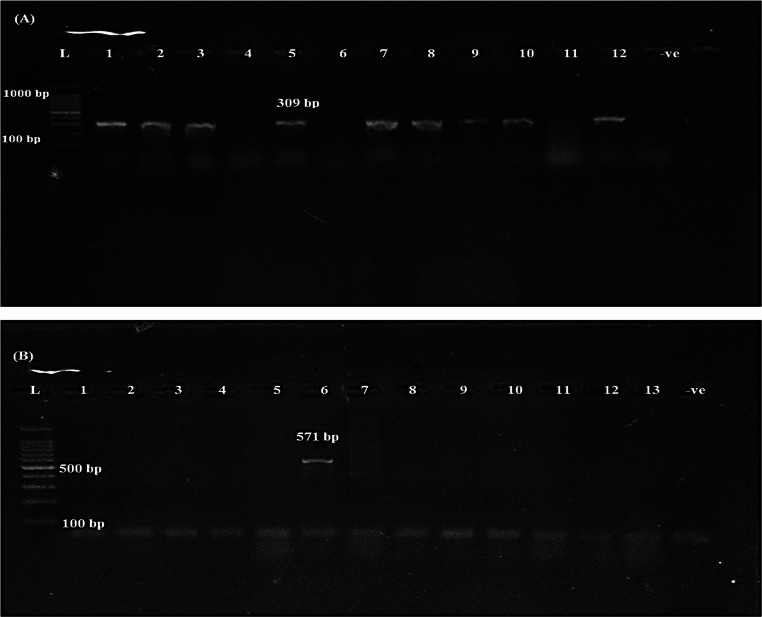
PCR amplification of virulence genes in *Salmonella* isolates. (**A**) *spi*C gene at 309 bp, (**B**) *spv*C gene at 571 bp. (L) 1000 bp DNA ladder; bp: base pairs

**Fig. 5:**
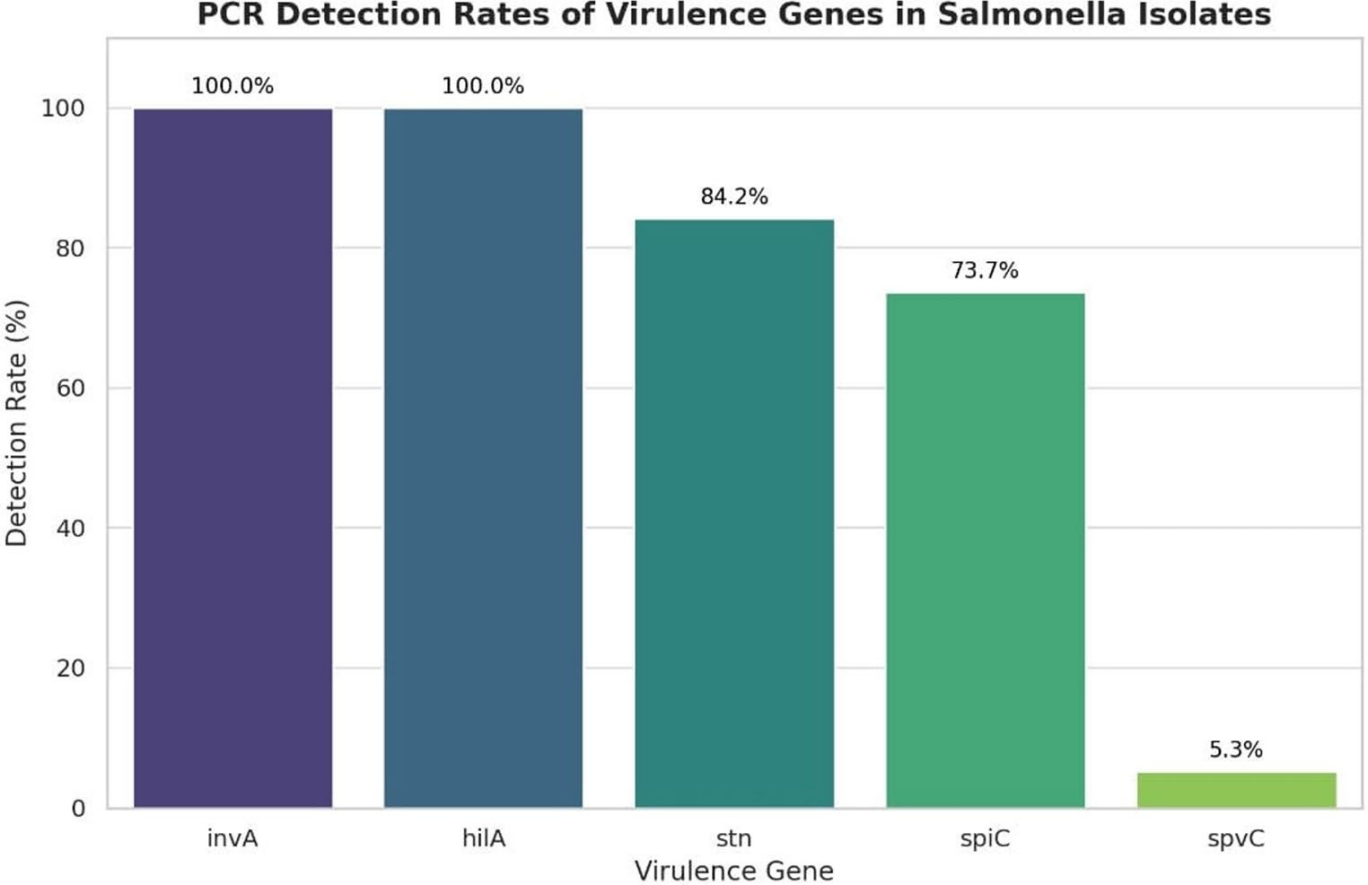
Detection rates of virulence genes by PCR among different *Salmonella* serovars. Each bar represents the percentage of isolates positive for each gene

**Fig. 6:**
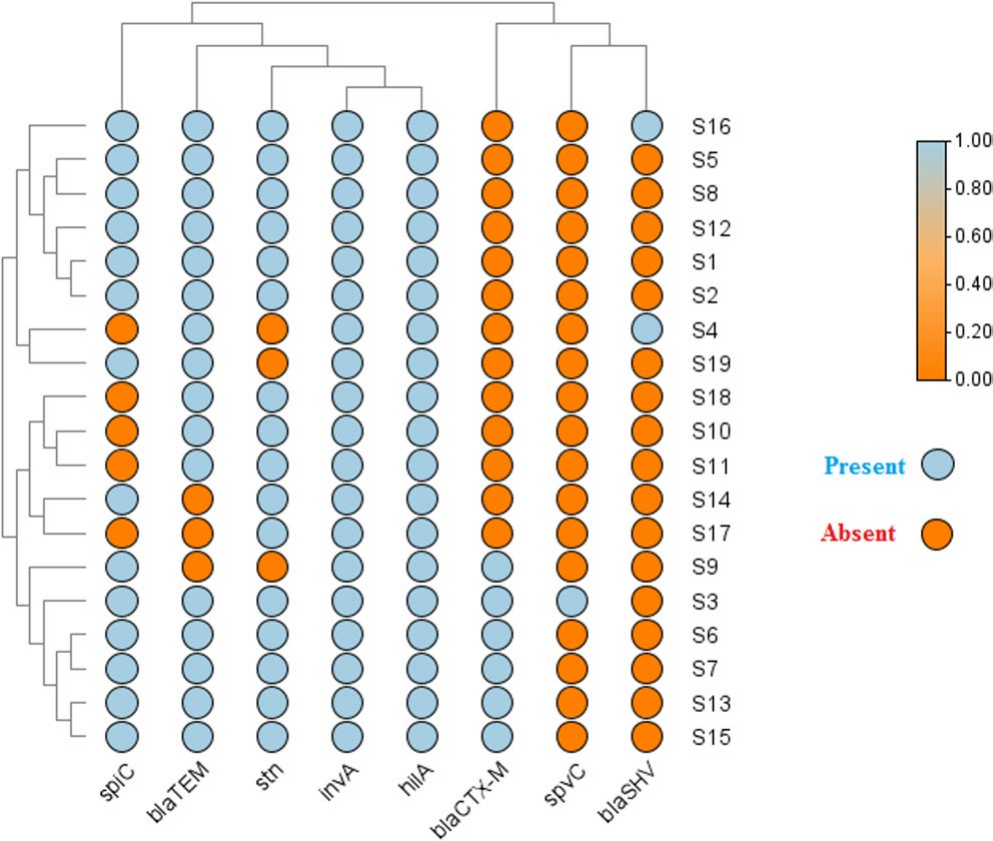
Heatmap showing the distribution of virulence and ESBL genes across *Salmonella* serovars. Color intensity indicates the presence (blue) or absence (red) of each gene. Hierarchical clustering at the top and left groups similar isolates and genes based on their patterns

PCR amplification was employed to identify specific virulence-associated genes in the *Salmonella* isolates. Both *inv*A and *hil*A genes were consistently detected across all examined isolates. (Fig. 3A), with a detection rate of 100% (19/19), indicating their conserved presence among *Salmonella* strains. The *stn* gene was identified in 84.21% (16/19) of the isolates (Fig. 3B), while the *spi*C gene was present in 73.68% (14/19) of the isolates (Fig. 4A). In contrast, the *spv*C gene showed a low prevalence and was detected in only 5.26% (1/19) of the isolates (Fig. 4B) (Figs. 5 and 6).

### Antimicrobial susceptibility testing of Salmonella serovars

**Fig. 7:**
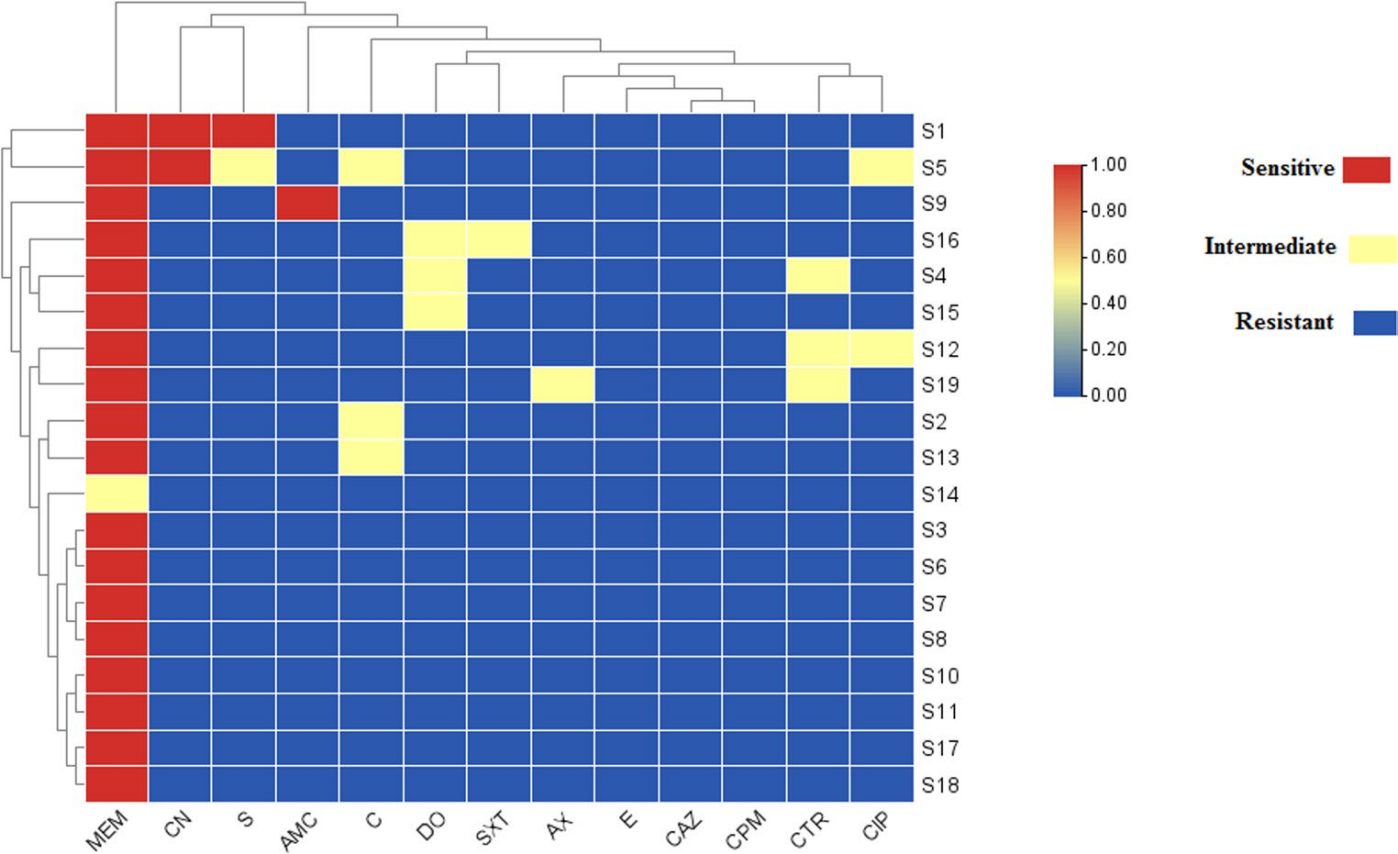
Heatmap of antimicrobial resistance profiles. Colors represent resistance status: blue = resistance, yellow = intermediate, and red = sensitivity. The clustering patterns suggest groups of isolates with similar resistance phenotypes, potentially linked to shared genetic backgrounds or common resistance determinants

The result of antimicrobial susceptibility testing of all *Salmonella* serovars (*n* = 19) revealed that all isolates were completely resistant (100%) to Ceftazidime (CAZ, 30 µg), Cefepime (CPM, 30 µg), and Erythromycin (E, 15 µg). High resistance was also observed against Amoxicillin (AX, 25 µg) (94.7%), Amoxicillin-clavulanic acid (AMC, 30 µg) (94.7%), Ceftriaxone (CTR, 30 µg) (84.2%), Chloramphenicol (C, 30 µg) (84.2%), Doxycycline (DO, 30 µg) (84.2%), Ciprofloxacin (CIP, 5 µg) (89.5%), Gentamicin (CN, 10 µg) (89.5%), Streptomycin (S, 10 µg) (89.5%), and Trimethoprim/sulfamethoxazole (SXT, 25 µg) (94.7%). In contrast, a high level of sensitivity was observed only with meropenem (MEM, 10 µg), where 94.7% of the isolates were susceptible (Fig. 7; Table 3). Based on the antibiogram profiles of the tested *Salmonella* isolates, resistance types were categorized into multidrug-resistant (MDR) and extensively drug-resistant (XDR). Notably, extensively drug-resistant (XDR) phenotypes were identified in 18 isolates (94.7%). In addition, one isolate (5.3%) was classified as multidrug-resistant (MDR). A total of ten distinct antimicrobial resistance profiles were observed, with the most frequent profile (*n* = 9) including AX, AMC, CTR, CAZ, CPM, C, DO, CIP, E, CN, S, and SXT. MDRI values among the tested isolates ranged from 0.7 to 0.9 **(**Table 4).

**Table 3:** Percentage of antimicrobial susceptibility for *Salmonella* serovars

Antimicrobial agent	Family	Disc code	CPD	*Salmonella* serovars *n* = 19					
				Resistance		Intermediate		Sensitive	
				No	%	No	%	No	%
Amoxicillin	β-lactams (Aminopenicillins)	AX	25 µg	**18**	**94.7**	**1**	**5.3**	0	0.0
Amoxicillin-clavulanic acid	Penicillin + β-lactamase inhibitor	AMC	30 µg	**18**	**94.7**	0	0.0	1	**5.3**
Ceftriaxone	Cephalosporin	CTR	30 µg	**16**	**84.2**	**3**	1**5.8**	0	0.0
Ceftazidime		CAZ	30 µg	**19**	100	0	0.0	0	0.0
Cefepime		CPM	30 µg	**19**	100	0	0.0	0	0.0
Meropenem	Carbapenems	MEM	10 µg	0	0.0	1	**5.3**	**18**	**94.7**
Chloramphenicol	Phenicol	C	30 µg	**16**	**84.2**	3	1**5.8**	0	0.0
Doxycycline	Tetracyclines	DO	30 µg	**16**	**84.2**	**3**	1**5.8**	**0**	3.4
Ciprofloxacin	Fluoroquinolones	CIP	5 µg	**17**	**89.5**	2	**10.5**	0	0.0
Erythromycin	Macrolides	E	15 µg	**19**	100	0	0.0	0	0.0
Gentamicin	Aminoglycoside	CN	10 µg	**17**	**89.5**	**0**	**0.0**	**2**	1**0.5**
Streptomycin		S	10 µg	**17**	**89.5**	1	**5.3**	1	**5.3**
DHFR inhibitor + Sulfonamides	DHFR inhibitor + Sulfonamides	SXT	25 µg	**18**	**94.7**	1	**5.3**	0	0.0

*CPD* concentration of the paper disc, *n* number of isolates

**Table 4:** Resistance profiles and antibiotypes identified in *Salmonella* serovars

Antibiotypes	Resistance profiles	No. of isolates	No. of resistant antibiotics	MDRINDEX	Resistance classification
I	AX, AMC, CTR, CAZ, CPM, C, DO, CIP, E, CN, S, SXT	9	12/13	0.9	XDR
II	AX, AMC, CTR, CAZ, CPM, DO, CIP, E, CN, S, SXT	2	11/13	0.8	XDR
III	AX, CTR, CAZ, CPM, C, DO, CIP, E, CN, S, SXT	1	11/13	0.8	XDR
IV	AX, AMC, CTR, CAZ, CPM, C, CIP, E, CN, S, SXT	1	11/13	0.8	XDR
V	AX, AMC, CTR, CAZ, CPM, C, DO, CIP, E, SXT	1	10/13	0.8	XDR
VI	AX, AMC, CAZ, CPM, C, CIP, E, CN, S, SXT	1	10/13	0.8	XDR
VII	AX, AMC, CAZ, CPM, C, DO, E, CN, S, SXT	1	10/13	0.8	XDR
VIII	AX, AMC, CTR, CAZ, CPM, C, CIP, E, CN, S	1	10/13	0.8	XDR
IX	AMC, CAZ, CPM, C, DO, CIP, E, CN, S, SXT	1	10/13	0.8	XDR
X	AX, AMC, CTR, CAZ, CPM, DO, E, CN, SXT	1	9/13	0.7	MDR

*AX* Amoxicillin, *AMC* Amoxicillin-clavulanic acid, *CTR* Ceftriaxone, *CAZ* Ceftazidime, *CPM* Cefepime, *C* Chloramphenicol, *DO* Doxycycline, *CIP* Ciprofloxacin, *E* Erythromycin, *CN* Gentamicin, *S* Streptomycin, *SXT* DHFR inhibitor + Sulfonamides. MDRINDEX = number of resistant antibiotics/total tested (13). MDR = multidrug-resistant, XDR = extensively drug-resistant

### Detection of ESBL-encoding genes

**Fig. 8:**
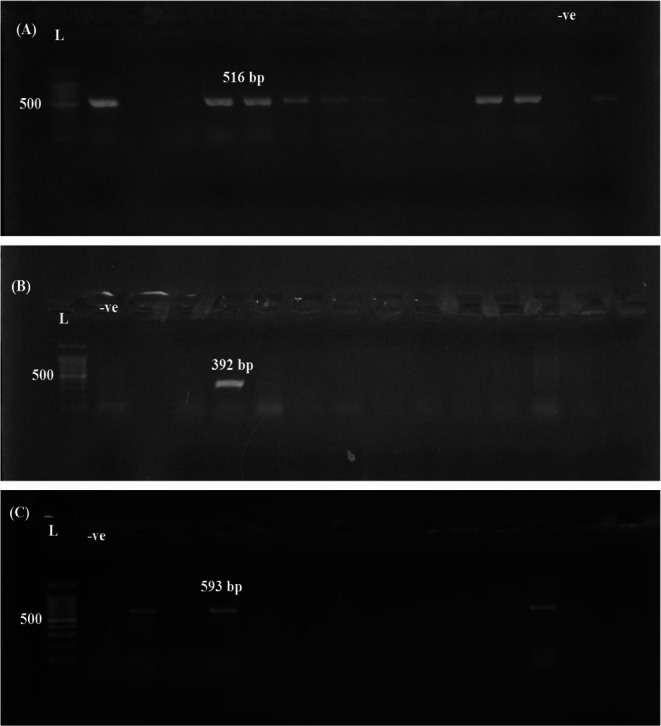
PCR amplification of ESBL genes in *Salmonella* isolates. (**A**) *bla*_TEM_ gene at 516 bp, (**B**) *bla*_SHV_ gene at 392 bp, (**C**) *bla*_CTX−M_ gene at 593 bp. (L) 1000 bp DNA ladder; bp: base pairs

Molecular screening for ESBL genes revealed a high prevalence of *bla*_TEM_, which was detected in 16 out of 19 isolates (84.2%). The *bla*_*CTX−M*_ gene was identified in 6 isolates (31.6%), while *bla*_SHV_ was present in only 2 isolates (10.5%) (Figs. 6 and 8).

### Correlation analysis between antimicrobial resistance genes and phenotypic resistance

**Fig. 9:**
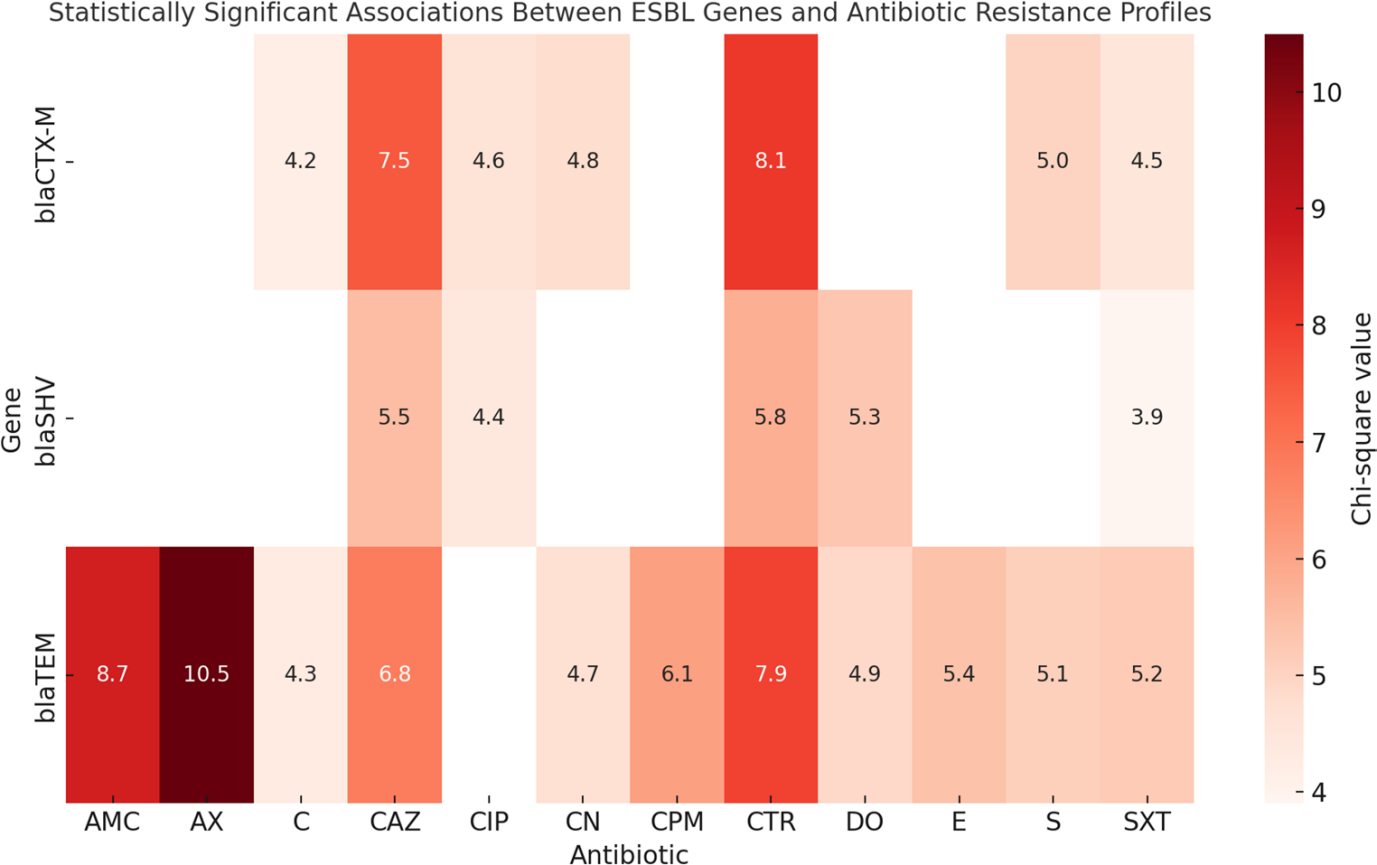
Heatmap illustrating statistically significant associations between ESBL genes and resistance to multiple antimicrobial agents among *Salmonella* isolates. Color intensity represents the strength of association, with darker colors indicating stronger associations

The study demonstrated a strong association between ESBL genes and phenotypic β-lactam resistance in *Salmonella* isolates. The *bla*_TEM_ gene was predominant and linked to resistance against multiple β-lactams, including amoxicillin-clavulanic acid, amoxicillin, ceftriaxone, ceftazidime, and cefepime. The *bla*_SHV_ gene was rare but found in multidrug-resistant isolates. The *bla*_CTX−M_ gene was associated with high resistance to third- and fourth-generation cephalosporins and often co-occurred with *bla*_TEM_, contributing to extensively drug-resistant phenotypes. Some isolates showed β-lactam resistance without ESBL genes. Additionally, isolates harboring *bla*_TEM_ and/or *bla*_CTX−M_ exhibited elevated resistance rates to non-β-lactam antibiotics, including tetracycline, ciprofloxacin, chloramphenicol, erythromycin, and trimethoprim–sulfamethoxazole (Fig. 9; Table 5).

### Association between ESBL-encoding genes and virulence determinants

**Fig. 10:**
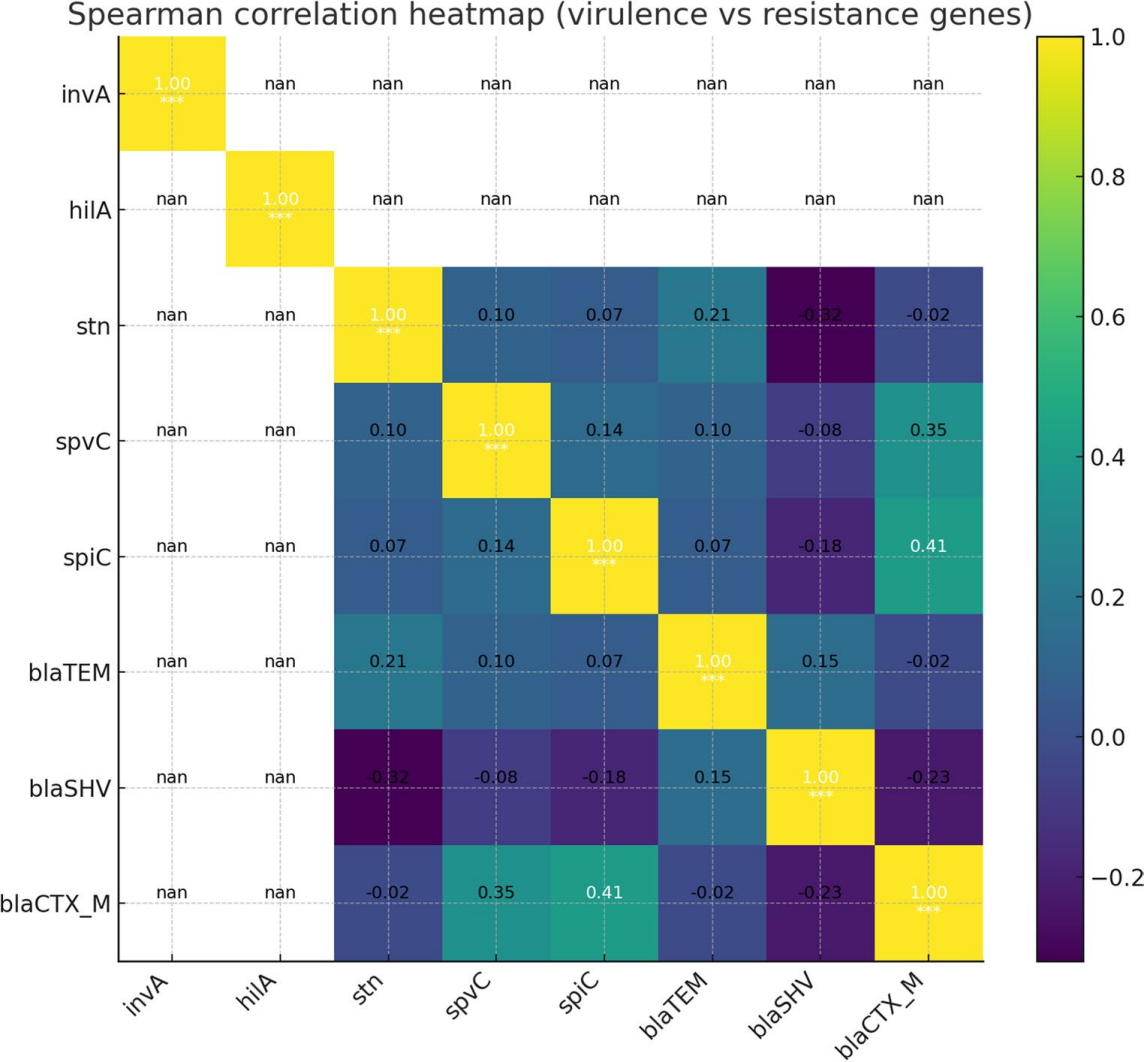
Spearman rank-correlation analysis between virulence genes and ESBL genes among *Salmonella* isolates (*n* = 19). Lighter yellow shades indicate stronger correlations between the presence of virulence genes and ESBL genes, while darker shades indicate weaker correlations

Analysis of distribution patterns showed that ESBL genes, particularly *bla*_TEM_ and *bla*_CTX−M_, frequently co-occurred with virulence genes *inv*A, *hil*A, and *stn* in *Salmonella* isolates. All isolates tested were positive for *inv*A and *hil*A, precluding correlation analysis for these loci. Spearman rank-correlation analysis revealed the strongest positive association between *spi*C and *bla*_CTX−M_ (ρ = +0.41), although this association did not reach statistical significance (*p* = 0.085). Other correlations between ESBL genes and virulence markers were weak and non-significant (e.g., *stn* vs. *bla*_SHV_, ρ = −0.32, *p* = 0.18; *bla*_TEM_ vs. *stn*, ρ = +0.21, *p* = 0.39) (Fig. 10).

**Table 5:** Association between ESBL genes and antibiotic resistance profiles (Chi-square test)

Gene	Antibiotic	χ² value	*p*-value	Scientific Level	Interpretation
*bla*_TEM_	AX	~ 10.5	0.001	Highly significant	Strong association between *bla*_TEM_ and resistance to amoxicillin
*bla*_TEM_	AMC	~ 8.7	0.003	Highly significant	Strong association with amoxicillin–clavulanic acid resistance
*bla*_TEM_	CTR	~ 7.9	0.005	Highly significant	Strong association with cefotaxime resistance
*bla*_TEM_	CAZ	~ 6.8	0.009	Highly significant	Strong association with ceftazidime resistance
*bla*_TEM_	CPM	~ 6.1	0.013	Significant	Significant association with cefepime resistance
*bla*_TEM_	DO	~ 4.9	0.026	Significant	Significant association with tetracycline resistance
*bla*_TEM_	E	~ 5.4	0.021	Significant	Significant association with erythromycin resistance
*bla*_TEM_	S	~ 5.1	0.024	Significant	Significant association with streptomycin resistance
*bla*_TEM_	CN	~ 4.7	0.031	Significant	Significant association with gentamicin resistance
*bla*_TEM_	C	~ 4.3	0.038	Significant	Significant association with chloramphenicol resistance
*bla*_TEM_	SXT	~ 5.2	0.023	Significant	Significant association with trimethoprim–sulfamethoxazole resistance
*bla*_CTX−M_	CTR	~ 8.1	0.004	Highly significant	Strong association with cefotaxime resistance
*bla*_CTX−M_	CAZ	~ 7.5	0.006	Highly significant	Strong association with ceftazidime resistance
*bla*_CTX−M_	CIP	~ 4.6	0.032	Significant	Significant association with ciprofloxacin resistance
*bla*_CTX−M_	S	~ 5.0	0.025	Significant	Significant association with streptomycin resistance
*bla*_CTX−M_	CN	~ 4.8	0.029	Significant	Significant association with gentamicin resistance
*bla*_CTX−M_	C	~ 4.2	0.040	Significant	Significant association with chloramphenicol resistance
*bla*_CTX−M_	SXT	~ 4.5	0.034	Significant	Significant association with trimethoprim-sulfamethoxazole resistance
*bla*_SHV_	CTR	~ 5.8	0.016	Significant	Significant association with cefotaxime resistance
*bla*_SHV_	CAZ	~ 5.5	0.019	Significant	Significant association with ceftazidime resistance
*bla*_SHV_	DO	~ 5.3	0.021	Significant	Significant association with tetracycline resistance
*bla*_SHV_	CIP	~ 4.4	0.036	Significant	Significant association with ciprofloxacin resistance
*bla*_SHV_	SXT	~ 3.9	0.048	Borderline significant	Possible association with trimethoprim-sulfamethoxazole resistance

χ²: Chi-square value; p-value: probability value; Scientific Level indicates statistical significance; Antibiotics abbreviations: AX = Amoxicillin, AMC = Amoxicillin-clavulanic acid, CTR = Ceftriaxone, CAZ = Ceftazidime, CPM = Cefepime, DO = Doxycycline, E = Erythromycin, CN = Gentamicin, S = Streptomycin, C = Chloramphenicol, SXT = Trimethoprim–sulfamethoxazole

## Discussion

*Salmonella enterica* is a major zoonotic pathogen in poultry, posing a risk to human health (Shaji et al. 2023). In this study, PCR detection of the *inv*A gene showed a prevalence of 9.5%, with 68.4% of isolates from internal organs and 31.6% from cloacal swabs, confirming previous reports that organ samples yield higher isolation rates (Williams and Athrey 2020).

Our findings of 9.5% *Salmonella* prevalence align with several studies in Egypt. Tawfik et al. (2022) reported 8% in broiler farms, Abd El-Mohsen et al. (2022) 11.36%, and Algammal et al. (2023) 9.3% in diseased broilers, mainly from cloacal swabs, whereas in our study internal organs showed higher prevalence, suggesting differences in sampling and infection dynamics. Orabi et al. (2022) recovered 10% from broilers. Higher prevalence was reported by Shalaby et al. (2022, 36.5%), Elsayed et al. (2023, 17%) in small-scale farms, and Tawakol et al. (2024, 28.4%) in migratory birds and farm environments. Lower rates were observed by Elfeil et al. (2020, 6.95%), El-Sendiony et al. (2021, 6.0%), and Rabie et al. (2023, 6.6%). These studies indicate the persistent circulation of *Salmonella* in Egypt, with variations likely due to sampling methods, farm management, biosecurity, and geographic factors.

To reduce *Salmonella* prevalence on poultry farms, comprehensive control strategies are needed across the production chain. Key measures include strict biosecurity, proper disinfection, controlled personnel and vehicle movement, improved farm hygiene, effective waste management, and vaccination against prevalent serovars. Additionally, the use of probiotics, routine monitoring of flocks and the environment, and training farm personnel on hygiene and responsible antimicrobial use are crucial to limit bacterial spread and the emergence of resistant strains (Neelawala et al. 2024).

Serotyping of *Salmonella* is crucial for epidemiological surveillance, outbreak investigations, and risk assessment. The Kauffmann-White scheme currently recognizes over 2,600 serovars, each differing in host specificity, virulence, disease manifestation, and geographic distribution (Tang et al. 2019; Han et al. 2024).

Serological typing of the 19 *Salmonella* isolates revealed four distinct serovars: *S*. Kentucky (10 isolates), *S*. Typhimurium (4), *S*. Salamae (3), and *S*. Infantis (2). The serotypes identified in the current study are consistent with earlier findings in Egypt; studies by Awad et al. (2020), El-Sherry (2022), Moawad et al. (2022), Orabi et al. (2022), Algammal et al. (2023), and Tawakol et al. (2024) reported recovery of *S*. Kentucky, *S*. Typhimurium, *S*. Infantis, and *S*. Salamae from poultry.

The predominance of *S*. Kentucky is particularly noteworthy and concerning. This serovar has been frequently associated with multidrug resistance and poses a significant zoonotic risk, especially in poultry production systems. Its high prevalence in the current study aligns with previous reports from Egypt and worldwide, indicating an increasing trend in its emergence as a major foodborne pathogen with enhanced resistance traits (Wang et al. 2023). The presence of *S*. Typhimurium, a well-known serovar implicated in human salmonellosis outbreaks, further underscores the public health risk associated with the isolates (Serrano-Fujarte et al. 2024).

Although *S.* Infantis and *S.* Salamae were identified at lower frequencies, both serovars have been associated with poultry and food sources. *S.* Infantis is a well-documented foodborne pathogen frequently isolated from chicken meat and eggs, and it has been implicated in several outbreaks of human gastroenteritis worldwide (Zhao et al. 2021). In contrast, *S.* Salamae is considered a non-typhoidal *Salmonella* subspecies that is less commonly involved in human infections. However, it has been occasionally isolated from poultry and food products and may exhibit pathogenic potential under certain conditions, especially in immunocompromised individuals (Hounmanou et al. 2022).

The diversity of the clinically and epidemiologically significant serotypes observed in the current study suggests multiple contamination sources and possible persistence of different *Salmonella* clones within the poultry environment. These findings highlight the importance of routine serotyping and molecular surveillance to track epidemiological trends and guide targeted intervention strategies in the poultry sector. It is important to note that the serotypes identified in this study do not represent the full spectrum of *Salmonella* serovars circulating in poultry. Other serotypes may also be present but were not detected, as the distribution of specific *Salmonella* serotypes varies between regions and countries. This variation is influenced by several factors, including geographical location, environmental conditions, farming practices, and the movement of poultry between regions. Furthermore, the spread of these serotypes across borders can occur through international trade, the migration of infected birds, or contaminated feed, thereby complicating efforts to control their transmission (Perilli et al. 2022).

Virulence genes play a crucial role in *Salmonella* pathogenicity. Their detection provides insights into virulent strains in poultry and guides preventive strategies, including biosecurity, vaccination, and effective risk assessment (Fàbrega and Vila 2013; Abdel Rahman et al. 2017; Shaji et al. 2023).

PCR screening in this study showed that all 19 *Salmonella* isolates carried *inv*A and *hil*A (100%). The *stn* gene was present in 84.2% of isolates, *spi*C in 73.7%, and *spv*C in only one isolate (5.3%). These findings are consistent with previous studies in Egypt, though gene prevalence varied by serotype, source, and region. For instance, Ammar et al. (2016) reported *inv*A in 100%, *hil*A in 88.2%, *stn* in 58.8%, and *spv*C in 5.9% of broiler isolates; Awad et al. (2020) found *inv*A and *stn* in 100% of retail chicken meat isolates; Elhariri et al. (2020) observed *inv*A (100%), *stn* and *spiC* (72.8%), *spv*C (66.7%), and hilA (3%) in *S*. Heidelberg from broilers and workers; Youssef et al. (2021) detected *inv*A in 100%, with *spi*A, stn, and *spv*C at 82%, 74%, and 24%, respectively; Diab et al. (2023) found *inv*A in 100%, *hil*A in 94.4%, *stn* in 72.2%, and *spv*C in 30.6%; and Algammal et al. (2023) reported *inv*A, *stn*, and *hil*A in *S*. Enteritidis (100%, 100%, 90%) and *S*. Typhimurium (100%, 100%, 93.3%). Overall, *inv*A consistently appears as the most prevalent and conserved virulence gene, highlighting its diagnostic importance, whereas *spv*C shows lower and more variable prevalence.

The low prevalence of the *spv*C gene in this study likely reflects its plasmid-borne nature, as it is not chromosomally encoded and is typically found in a limited number of serovars. Here, *spv*C was detected only in *S*. Typhimurium, consistent with reports that the *spv* plasmid occurs mainly in *S*. Typhimurium, *S*. Enteritidis, and *S*. Dublin (Guiney and Fierer 2011). The predominance of *S*. Kentucky, which usually lacks this plasmid, explains the overall low detection. Additionally, environmental factors or plasmid loss during sub-culturing and sample handling may contribute to its reduced prevalence (Silva et al. 2017).

The detection of the *stn* gene in the examined *Salmonella* isolates is concerning, as it encodes the *Salmonella* enterotoxin, a key virulence factor causing diarrheal illness. Its presence indicates the pathogenic potential of the isolates and their ability to induce gastrointestinal symptoms such as diarrhea, abdominal cramps, and fever, posing a public health risk, especially for children, the elderly, and immunocompromised individuals (Nakano et al. 2012).

The detection of these virulent profiles highlights the importance of ongoing molecular surveillance and rigorous biosecurity measures in poultry production. Such data are valuable for risk assessment and help guide targeted strategies to prevent the spread of virulent *Salmonella* strains, thereby safeguarding both animal and public health (Lozano-Villegas et al. 2023).

Antimicrobial resistance in *Salmonella* is a major threat to animal and human health. Extensive antibiotic use in poultry has led to multidrug- and extensively drug-resistant strains, which can be transmitted to humans, causing difficult-to-treat infections (Salam et al. 2023). The antimicrobial susceptibility testing revealed a concerning resistance pattern among the *Salmonella* isolates in this study. These findings reflect a significant challenge in treating *Salmonella* infections using commonly prescribed antibiotics, particularly β-lactams and aminoglycosides. In contrast, meropenem (MEM) remained highly effective, with 94.7% of isolates showing susceptibility, highlighting its potential as a last-resort treatment option. However, the reliance on carbapenems is concerning, given their critical importance in human medicine and the risk of emerging carbapenem-resistant strains.

The antimicrobial resistance profiles observed in this study align with previous reports from Egypt, reflecting the widespread occurrence of resistant *Salmonella* in poultry. Awad et al. (2020) reported high resistance to erythromycin (96.78%), trimethoprim–sulfamethoxazole (93.55%), doxycycline (93.55%), streptomycin (80.65%), and amoxicillin (67.8%), comparable to our findings. Similarly, Youssef et al. (2021) observed complete resistance to amoxicillin and erythromycin and high resistance to doxycycline (94%). Shaheen et al. (2024) also reported elevated resistance to streptomycin (53.33%), trimethoprim–sulfamethoxazole (83.33%), and amoxicillin–clavulanic acid (90%). Overall, these studies corroborate our data, particularly the high resistance to β-lactams, macrolides, and tetracyclines.

The antimicrobial resistance patterns observed in this study, including a high prevalence of XDR phenotypes (94.7%) and multidrug resistance across multiple antibiotic classes, align with recent reports from Egypt. Elsayed et al. (2024) documented widespread MDR *S*. Enteritidis with notable genetic diversity in broiler and layer farms, while Algammal et al. (2023) characterized XDR *S*. Enteritidis and *S*. Typhimurium isolates. Together, these studies highlight the alarming spread of MDR and XDR *Salmonella* in Egyptian poultry, supporting our findings.

The consistent resistance profiles across these studies highlight the ongoing misuse and overuse of antibiotics in the poultry sector. This widespread multidrug resistance underscores the need for routine surveillance, strict regulatory policies, and integrated One Health approaches to limit the dissemination of resistant *Salmonella* along the food chain and mitigate associated public health risks.

The emergence of extended-spectrum β-lactamase (ESBL) in poultry represents a significant One Health concern, as it bridges the gap between animal reservoirs and human infections (Dandachi et al. 2018; Widodo et al. 2024). Our findings revealed a high prevalence of *bla*_TEM_ among the *Salmonella* isolates (84.2%), followed by *bla*_CTX−M_ (31.6%) and *bla*_SHV_ (10.5%). These results are broadly consistent with previous reports from Egypt. For instance, Adel et al. (2021) detected *bla*_TEM−1_ in 79.4% of isolates, *bla*_CTX−M_ in 32.4%, and *bla*_SHV−12_ in 14.7%. Similarly, Kamel et al. (2021) reported the presence of *bla*_TEM_ in 100% of tested isolates and *bla*_SHV_ in 20%, while *bla*_CTX−M_ was entirely absent. Orabi et al. (2022) reported that chicken isolates harbored *bla*_TEM_ and bla_CTX_ at 100% and 11.1%. In another recent study, El-Saeed et al. (2024) detected *bla*_TEM_ in 21 isolates and *bla*_CTX−M−1_ in 16, further supporting the predominance of *bla*_TEM_ among poultry-associated *Salmonella* strains in Egypt.

The high prevalence of the *bla*_TEM_ gene (84.2%) among our *Salmonella* isolates likely reflects its early emergence and widespread dissemination in both clinical and environmental reservoirs. As one of the first identified ESBL genes, *bla*_TEM_ is commonly found in Gram-negative bacteria, including *Salmonella enterica*, particularly in developing countries (Rawat and Nair 2010). Its association with various plasmids and transposable elements facilitates horizontal gene transfer, supporting its persistent presence in animal and human microbiota (Bielak et al. 2011).

The higher detection of *bla*_CTX−M_ (31.6%) compared to *bla*_SHV_ (10.5%) in our *Salmonella* isolates aligns with global trends, as CTX-M enzymes have become the dominant ESBL type over the past two decades (Bevan et al. 2017). Frequently linked to mobile genetic elements such as IncI1 and IncF plasmids, *bla*_CTX−M_ spreads rapidly in food-producing animals, partly due to the selective pressure from third-generation cephalosporin use. In contrast, *bla*_SHV_ is more common in nosocomial *Klebsiella pneumoniae* and less frequent in non-clinical *Salmonella* isolates, explaining its lower prevalence in our study (Liakopoulos et al. 2016; Rossolini et al. 2008).

The relatively high occurrence of these ESBL genes suggests ongoing dissemination of β-lactam resistance determinants within the poultry production sector. Such findings underscore the importance of implementing molecular surveillance strategies to monitor the spread of ESBL-producing *Salmonella* and guide appropriate antimicrobial stewardship interventions.

The present study revealed a clear association between ESBL genes and phenotypic resistance to β-lactam antibiotics among the *Salmonella* isolates. The *bla*_TEM_ gene was frequently accompanied by resistance to a broad range of β-lactams, particularly amoxicillin-clavulanic acid, amoxicillin, ceftriaxone, ceftazidime, and cefepime. These findings reinforce the established role of *bla*_TEM_ in mediating resistance to both β-lactam/β-lactamase inhibitor combinations and extended-spectrum cephalosporins. In contrast, *bla*_SHV_ was rare (10.5%), observed in only two isolates that exhibited resistance to most β-lactams, suggesting a potential additive effect when present. Notably, *bla*_SHV_ -positive isolates generally exhibited a multidrug-resistant (MDR) phenotype, although its low frequency. The *bla*_CTX−M_ gene displayed marked resistance to third- and fourth-generation cephalosporins, consistent with its known functional profile. When co-occurring with *bla*_TEM_, *bla*_CTX−M_ appeared to enhance resistance levels, sometimes contributing to extensively drug-resistant (XDR) patterns.

Notably, several isolates exhibited β-lactam resistance in the absence of these ESBL genes, indicating the possible involvement of alternative mechanisms such as AmpC β-lactamases, alterations in penicillin-binding proteins (PBPs), or efflux pump overexpression. These results highlight the multifactorial nature of β-lactam resistance in *Salmonella* and underscore the importance of comprehensive genomic surveillance to fully elucidate the resistance determinants circulating in poultry-associated strains.

The resistance beyond β-lactam antibiotics revealed that high resistance rates to tetracycline (DO), ciprofloxacin (CIP), chloramphenicol (C), erythromycin (E), and trimethoprim–sulfamethoxazole (SXT) were observed among isolates carrying *bla*_TEM_ and/or *bla*_CTX−M_. This pattern suggests possible co-location of resistance genes on the same plasmids or co-selection mechanisms. The detection of such associations in non–β-lactam classes supports the hypothesis of mobile genetic elements carrying multiple resistance determinants.

Increasing evidence indicates that ESBL-encoding genes and virulence-associated genes can co-localize on the same mobile genetic elements, such as plasmids and integrons. For example, plasmids carrying *bla*_CTX−M_ have been reported to also harbor virulence genes like *spv*C and *hil*A, suggesting that the acquisition of resistance may occur alongside virulence (Guo and Zhao 2021). This co-occurrence can enhance bacterial fitness by allowing strains to withstand antimicrobial pressure while maintaining or even amplifying pathogenicity. The simultaneous presence of virulence genes and antibiotic resistance in *Salmonella* isolates represents a serious public health threat, as multidrug-resistant strains with virulence traits increase the risk of treatment failure, complicate clinical management, and promote persistence in the environment and food chain. Continuous monitoring of both virulence profiles and resistance patterns is therefore essential to control the spread of pathogenic, drug-resistant *Salmonella* strains.

Comparative analysis revealed a strong association between ESBL-encoding genes, particularly *bla*_TEM_ and *bla*_CTX−M_, and key virulence genes in *Salmonella* isolates, including *inv*A, *hil*A, *stn*, *spv*C, and *spi*C. Most ESBL-positive isolates co-harbored invasion-related genes (*inv*A and *hil*A) and the enterotoxin gene *stn*, showing that acquiring antibiotic resistance did not reduce their virulence. Notably, isolates carrying *bla*_CTX−M_ often possessed *spi*C, which enhances intracellular survival, suggesting a potential increase in pathogenicity. Even the less frequent *bla*_SHV_ was commonly found alongside multiple virulence genes, indicating that rare ESBL variants can also contribute to highly virulent strains. These findings highlight the likelihood of co-selection and genetic linkage between antimicrobial resistance and virulence traits, often mediated by mobile genetic elements, which may amplify the public health risk posed by multidrug-resistant *Salmonella*.

### Limitations

This study has some limitations. The relatively small sample size and restricted geographic coverage may limit the generalizability of the findings. In addition, only a limited set of resistance and virulence genes was investigated, without assessing their actual phenotypic expression. Whole-genome sequencing was not performed, which restricted further insights into plasmid-mediated resistance and clonal relatedness. Furthermore, pooling of visceral organs during sampling may have reduced the sensitivity of *Salmonella* detection in individual tissues; however, this approach was applied to increase sample representativeness and to reflect common diagnostic practices.

## Conclusions

In conclusion, the high prevalence of virulence genes among *Salmonella* isolates, along with their marked resistance to multiple antibiotics, highlights a growing concern in food safety and public health. The detection of Extended-Spectrum Beta-Lactamase (ESBL)-producing *Salmonella* further exacerbates this threat, as these enzymes confer resistance to critically important β-lactam antibiotics, limiting therapeutic options. These findings underscore the urgent need for integrated surveillance programs that monitor both virulence and resistance traits. Implementation of strict hygiene practices in poultry production, rational use of antimicrobials, and public awareness campaigns are essential steps toward reducing the risk of transmission of virulent, drug-resistant, and ESBL-producing *Salmonella* to humans through the food chain.

## Supplementary Information

Below is the link to the electronic supplementary material.

Supplementary Material 1 (DOCX 444 KB)

## Data Availability

The current study includes the entire information that was gathered throughout this manuscript. The data supporting the study conclusions are accessible from the authors upon request.

## References

[CR1] Abd El-Mohsen A, El-Sherry S, Soliman MA, Amen O (2022) Serological and antibacterial characteristics of *Salmonella* isolates from chickens in Assiut, Egypt. Benha Vet Med J 41(2):93–99. 10.21608/bvmj.2021.93816.1468

[CR2] Abdel Rahman MONA, Nasef S, Hamed E (2017) Detection of some virulence genes in *Salmonella* Kentucky isolated from poultry. Assiut Vet Med J 63(154):122–132

[CR3] Abreu R, Semedo-Lemsaddek T, Cunha E, Tavares L, Oliveira M (2023) Antimicrobial drug resistance in poultry production: current status and innovative strategies for bacterial control. Microorganisms 11(4):953. 10.3390/microorganisms11040953 37110376 10.3390/microorganisms11040953 PMC10141167

[CR4] Adel WA, Ahmed AM, Hegazy Y, Torky HA, Shimamoto T (2021) High prevalence of ESBL and plasmid-mediated quinolone resistance genes in *Salmonella enterica* isolated from retail meats and slaughterhouses in Egypt. Antibiotics 10(7):881. 10.3390/antibiotics10070881 34356803 10.3390/antibiotics10070881 PMC8300843

[CR5] Algammal AM, El-Tarabili RM, Abd El-Ghany WA et al (2023) Resistance profiles, virulence and antimicrobial resistance genes of XDR S. Enteritidis and S. Typhimurium. AMB Expr 13(1):110. 10.1186/s13568-023-01615-x 10.1186/s13568-023-01615-x PMC10564691 37817026

[CR6] Ammar AM, Mohamed AA, Abd El-Hamid MI, El-Azzouny MM (2016) Virulence genotypes of clinical *Salmonella* serovars from broilers in Egypt. J Infect Dev Ctries 10(4):337–346. 10.3855/jidc.6881 27130994 10.3855/jidc.7437

[CR7] Archambault M, Petrov P, Hendriksen RS, Asseva G, Bangtrakulnonth A, Hasman H, Aarestrup FM (2006) Molecular characterization and occurrence of extended-spectrum β-lactamase resistance genes among *Salmonella enterica* serovar corvallis from Thailand, Bulgaria, and Denmark. Microb Drug Resist 12(3):192–198. 10.1089/mdr.2006.12.192 17002546 10.1089/mdr.2006.12.192

[CR8] Awad A, Gwida M, Khalifa E, Sadat A (2020) Phenotypes, antibacterial-resistant profile, and virulence-associated genes of *Salmonella* serovars isolated from retail chicken meat in Egypt. Vet World 13(3):440. 10.14202/vetworld.2020.440-446 32367947 10.14202/vetworld.2020.440-445 PMC7183463

[CR9] Beceiro A, Tomás M, Bou G (2013) Antimicrobial resistance and virulence: a successful or deleterious association in the bacterial world? Clin Microbiol Rev 26(2):185–230. 10.1128/CMR.00059-12 23554414 10.1128/CMR.00059-12 PMC3623377

[CR10] Bevan ER, Jones AM, Hawkey PM (2017) Global epidemiology of CTX-M β-lactamases: temporal and geographical shifts in genotype. J Antimicrob Chemother 72(8):2145–2155. 10.1093/jac/dkx146 28541467 10.1093/jac/dkx146

[CR11] Bielak E, Bergenholtz RD, Jørgensen MS, Sørensen SJ, Hansen LH, Hasman H (2011) Investigation of diversity of plasmids carrying the blaTEM-52 gene. J Antimicrob Chemother 66(11):2465–2474. 10.1093/jac/dkr331 21831988 10.1093/jac/dkr331

[CR12] CLSI (2024) Performance standards for antimicrobial susceptibility testing. Clinical and Laboratory Standards Institute, Wayne, PA. https://www.darvashco.com/wp-content/uploads/2024/07/CLSI-2024_compressed-1.pdf

[CR13] Colom K, Pérez J, Alonso R, Fernández-Aranguiz A, Lariño E, Cisterna R (2003) Simple and reliable multiplex PCR assay for detection of blaTEM, BlaSHV and blaOXA-1 genes in Enterobacteriaceae. FEMS Microbiol Lett 223(2):147–151. 10.1016/S0378- 12829279 10.1016/S0378-1097(03)00306-9

[CR14] Crump JA, Sjölund-Karlsson M, Gordon MA, Parry CM (2015) Epidemiology, clinical presentation, laboratory diagnosis, antimicrobial resistance, and antimicrobial management of invasive *Salmonella* infections. Clin Microbiol Rev 28(4):901–937. 10.1128/CMR.00002-15 26180063 10.1128/CMR.00002-15 PMC4503790

[CR15] Dandachi I, Sokhn ES, Dahdouh EA, Azar E, El-Bazzal B, Rolain JM, Daoud Z (2018) Prevalence and characterization of multidrug-resistant gram-negative bacilli isolated from Lebanese poultry: a nationwide study. Front Microbiol 9:550. 10.3389/fmicb.2018.00550 29628921 10.3389/fmicb.2018.00550 PMC5876231

[CR16] Diab MS, Thabet AS, Elsalam MA, Ewida RM, Sotohy SA (2023) Detection of virulence and β-lactamase resistance genes of non-typhoidal *Salmonella* isolates from human and animal origin in egypt: one health concern. Gut Pathog 15(1):16. 10.1186/s13099-023-00577-y 36998086 10.1186/s13099-023-00542-3 PMC10061834

[CR17] El-Saeed BA, Elshebrawy HA, Zakaria AI, Abdelkhalek A, Sallam KI (2024) Colistin-, cefepime-, and levofloxacin-resistant *Salmonella enterica* serovars isolated from Egyptian chicken carcasses. Ann Clin Microbiol Antimicrob 23(1):61. 10.1186/s12941-024-00713-3 38965586 10.1186/s12941-024-00713-3 PMC11229489

[CR18] El-Sendiony M, Kamar AM, Hussein DE (2021) Multi antibiogram-integron relationship in *Salmonella* species isolated from local poultry carcasses. Alex J Vet Sci 69(1):1–11. 10.5455/ajvs.58254

[CR19] El-Sherry S (2022) Serological and antibacterial characteristics of *Salmonella* isolates from chickens in Assiut, Egypt. Benha Vet Med J 41(2):93–99

[CR20] Elfeil WK, Ezzat ME, Fathi A, Alkilany MAA, Abouelmaatti RR (2020) Prevalence, genotypic analysis and antibiotic resistance of *Salmonella* species isolated from imported and freshly slaughtered chicken. Am J Anim Vet Sci 15:134–144. 10.3844/ajavsp.2020.134.144

[CR21] Elhariri M, Elhelw R, Selim S, Ibrahim M, Hamza D, Hamza E (2020) Virulence and antibiotic resistance patterns of extended-spectrum beta-lactamase-producing *Salmonella enterica* serovar Heidelberg isolated from broiler chickens and poultry workers: a potential hazard. Foodborne Pathog Dis 17(6):373–381. 10.1089/fpd.2019.2719 31755782 10.1089/fpd.2019.2719

[CR22] Elsayed MM, El-Basrey YFH, El-Baz AH (2024) Ecological prevalence, genetic diversity, and multidrug resistance of *Salmonella* enteritidis recovered from broiler and layer chicken farms. Poult Sci 103(2):103320. 10.1016/j.psj.2023.103320 38215504 10.1016/j.psj.2023.103320 PMC10825688

[CR23] European Food Safety Authority (EFSA) (2021) The European union summary report on antimicrobial resistance in zoonotic and indicator bacteria from humans, animals and food in 2018/2019. EFSA J 19(4):e06490. 10.2903/j.efsa.2021.6490 33868492 10.2903/j.efsa.2021.6490 PMC8040295

[CR24] Fàbrega A, Vila J (2013) *Salmonella enterica* serovar typhimurium skills to succeed in the host: virulence and regulation. Clin Microbiol Rev 26(2):308–341. 10.1128/CMR.00066-12 23554419 10.1128/CMR.00066-12 PMC3623383

[CR25] Guiney DG, Fierer J (2011) The role of the Spv genes in *Salmonella* pathogenesis. Front Microbiol 2:129. 10.3389/fmicb.2011.00129 21716657 10.3389/fmicb.2011.00129 PMC3117207

[CR26] Guo L, Zhao Y (2021) Global spread and molecular characterization of CTX-M-producing *Salmonella* typhimurium isolates. Antibiotics 10(11):1417. 10.3390/antibiotics10111417 34827355 10.3390/antibiotics10111417 PMC8614702

[CR27] Han J, Aljahdali N, Zhao S, Tang H, Harbottle H, Hoffmann M, Frye JG, Foley SL (2024) Infection biology of *Salmonella enterica*. EcoSal Plus 12(1):eesp00012023. 10.1128/ecosalplus.esp-0001-2023 38415623 10.1128/ecosalplus.esp-0001-2023 PMC11636313

[CR28] Hounmanou YMG, Baniga Z, García V (2022) *Salmonella* salamae and S. waycross isolated from Nile perch in Lake Victoria show limited human pathogenic potential. Sci Rep 12:4229. 10.1038/s41598-022-08200-5 35273308 10.1038/s41598-022-08200-5 PMC8913728

[CR29] Kamel NM, Farghaly EM, Shawky HM, Samir A (2021) Molecular characterisation of extended-spectrum β-lactamase-producing Escherichia coli and *Salmonella* isolated from poultry and poultry products in Egypt. Bulgarian J Vet Med 24(1):66–76. 10.15547/bjvm.2334

[CR30] Kong-Ngoen T, Santajit S, Tunyong W, Pumirat P, Sookrung N, Chaicumpa W, Indrawattana N (2022) Antimicrobial resistance and virulence of non-typhoidal *Salmonella* from retail foods marketed in Bangkok, Thailand. Foods 11(5):661. 10.3390/foods11050661 35267294 10.3390/foods11050661 PMC8909193

[CR31] Krzyzanowski F, Zappelini L, Martone-Rocha S et al (2014) Quantification and characterization of *Salmonella* spp. isolates in sewage sludge with potential usage in agriculture. BMC Microbiol 14:263. 10.1186/s12866-014-0263-x 25927729 10.1186/s12866-014-0263-x PMC4207900

[CR32] Liakopoulos A, Mevius D, Ceccarelli D (2016) A review of SHV extended-spectrum β-lactamases: neglected yet ubiquitous. Front Microbiol 7:1374. 10.3389/fmicb.2016.01374 27656166 10.3389/fmicb.2016.01374 PMC5011133

[CR33] Lozano-Villegas KJ, Herrera-Sánchez MP, Beltrán-Martínez MA, Cárdenas-Moscoso S, Rondón-Barragán IS (2023) Molecular detection of virulence factors in *Salmonella* serovars isolated from poultry and human samples. Vet Med Int 2023:1875253. 10.1155/2023/1875253 36910894 10.1155/2023/1875253 PMC9998162

[CR34] Magiorakos AP, Srinivasan A, Carey RB, Carmeli Y, Falagas ME, Giske CG, Monnet DL (2012) Multidrug-resistant, extensively drug-resistant and pandrug-resistant bacteria: an international expert proposal for interim standard definitions for acquired resistance. Clin Microbiol Infect 18(3):268–281. 10.1111/j.1469-0691.2011.03570.x 21793988 10.1111/j.1469-0691.2011.03570.x

[CR35] Markos T (2017) A review on pullorum disease and its economic importance in poultry industry. World’s Vet J 7(1):1–7. 10.5455/wvj.20170239

[CR36] Mashayekh Z, Moradi Bidhendi S, Khaki P (2022) Detection of invA, sivH, and AgfA virulence genes in *Salmonella* spp. isolated from broiler breeder farms in Alborz Province, Iran. Arch Razi Inst 77(2):607–614. 10.22092/ARI.2021.353674.1607 36284959 10.22092/ARI.2021.353674.1607 PMC9548246

[CR37] Moawad AA, Ammar AM, Rabie NS, Sherief MR, Eldesoukey IE (2022) Molecular characterization of *Salmonella enterica* serovars in broiler chickens at Kafr El-Sheikh governorate, Egypt. J Anim Plant Sci 32(6):1616–1623

[CR38] Nadi ZR, Salehi TZ, Tamai IA, Foroushani AR, Sillanpaa M, Dallal MMS (2020) Evaluation of antibiotic resistance and prevalence of common *Salmonella enterica* serovars isolated from foodborne outbreaks. Microchem J 155:104660. 10.1016/j.microc.2020.104660

[CR39] Nakano M, Yamasaki E, Ichinose A, Shimohata T, Takahashi A, Akada JK, Nakamura K, Moss J, Hirayama T, Kurazono H (2012) *Salmonella* enterotoxin (Stn) regulates membrane composition and integrity. Dis Model Mech 5(4):515–521. 10.1242/dmm.009324 22301710 10.1242/dmm.009324 PMC3380714

[CR40] Neelawala RN, Edison LK, Kariyawasam S (2024) Pre-harvest non-typhoidal *Salmonella* control strategies in commercial layer chickens. Animals 14(24):3578. 10.3390/ani14243578 39765482 10.3390/ani14243578 PMC11672659

[CR41] Odjadjare EC, Olaniran AO (2015) Prevalence of antimicrobial resistant and virulent *Salmonella* spp. in treated effluent and receiving aquatic milieu of wastewater treatment plants in Durban, South Africa. Int J Environ Res Public Health 12(8):9692–9713. 10.3390/ijerph120809692 26295245 10.3390/ijerph120809692 PMC4555307

[CR42] Orabi A, Armanious W, Radwan IA et al (2022) Genetic correlation of virulent *Salmonella* serovars (extended spectrum β-lactamases) isolated from broiler chickens and human: a public health concern. Pathogens 11(10):1196. 10.3390/pathogens11101196 36297253 10.3390/pathogens11101196 PMC9610193

[CR43] Perilli M, Scattolini S, Telera GC, Cornacchia A, Tucci P, Sacchini F, Sericola M, Romantini R, Marotta F, Di Provvido A, Pomilio F, De Massis F (2022) Distribution of *Salmonella* spp. serotypes isolated from poultry in Abruzzo and Molise regions (Italy) during a 6-year period. Microorganisms 10(2):199. 10.3390/microorganisms10020199 35208655 10.3390/microorganisms10020199 PMC8877327

[CR44] Rabie NS, Fedawy HS, Sedeek DM, Bosila MA, Abdelbaki MM, Ghetas AM, Samy AA (2023) Isolation and serological identification of current *Salmonella* species recovered from broiler chickens in Egypt. Egypt J Vet Sci 54(1):11–19. 10.21608/ejvs.2023.176482.1395

[CR45] Rahman MM, Hossain H, Chowdhury MSR, Hossain MM, Saleh A, Binsuwaidan R, Noreddin A, Helmy YA, El Zowalaty ME (2024) Molecular characterization of multidrug-resistant and extended-spectrum β-lactamases-producing *Salmonella enterica* serovars enteritidis and typhimurium isolated from raw meat in retail markets. Antibiotics 13(7):586. 10.3390/antibiotics13070586 39061268 10.3390/antibiotics13070586 PMC11274296

[CR46] Rawat D, Nair D (2010) Extended-spectrum β-lactamases in Gram-negative bacteria. J Glob Infect Dis 2(3):263–274. 10.4103/0974-777X.68531 20927289 10.4103/0974-777X.68531 PMC2946684

[CR47] Rossolini GM, D’Andrea MM, Mugnaioli C (2008) The spread of CTX-M-type extended-spectrum β-lactamases. Clin Microbiol Infect 14(Suppl 1):33–41. 10.1111/j.1469-0691.2007.01867.x 18154526 10.1111/j.1469-0691.2007.01867.x

[CR48] Salam MA, Al-Amin MY, Salam MT, Pawar JS, Akhter N, Rabaan AA, Alqumber MAA (2023) Antimicrobial resistance: a growing serious threat for global public health. Healthcare 11(13):1946. 10.3390/healthcare11131946 37444780 10.3390/healthcare11131946 PMC10340576

[CR49] Salem M, Awad A, Younis G (2023) Antibiotic susceptibility and molecular detection of virulent *Pseudomonas aeruginosa* isolated from bovine mastitis milk in Egypt. J Adv Vet Res 13(4):664–671

[CR50] Serrano-Fujarte I, Calva E, García-Domínguez J et al (2024) Population structure and ongoing microevolution of the emerging multidrug-resistant *Salmonella* typhimurium ST213. Npj Antimicrob Resist 2:10. 10.1038/s44259-024-00027-6 39843807 10.1038/s44259-024-00027-6 PMC11721120

[CR51] Shaheen R, El-Abasy M, El-Sharkawy H, Ismail MM (2024) Prevalence, molecular characterization, and antimicrobial resistance among *Escherichia coli*, *Salmonella* spp., and *Staphylococcus aureus* strains isolated from Egyptian broiler chicken flocks with omphalitis. Open Vet J 14(1):284. 10.5455/OVJ.2024.v14.i1.45 10.5455/OVJ.2024.v14.i1.25 PMC11018412 38633165

[CR52] Shaji S, Selvaraj RK, Shanmugasundaram R (2023) *Salmonella* infection in poultry: a review on the pathogen and control strategies. Microorganisms 11(11):2814. 10.3390/microorganisms11112814 38004824 10.3390/microorganisms11112814 PMC10672927

[CR53] Shalaby A, Ismail MM, El-Sharkawy H (2022) Isolation, identification, and genetic characterization of antibiotic resistance of *Salmonella* species isolated from chicken farms. J Trop Med 2022:6065831. 10.1155/2022/6065831 36482931 10.1155/2022/6065831 PMC9726267

[CR54] Silva C, Puente JL, Calva E (2017) *Salmonella* virulence plasmid: pathogenesis and ecology. Pathog Dis 75(6):ftx070. 10.1093/femspd/ftx070 10.1093/femspd/ftx070 28645187

[CR55] Tang S, Orsi RH, Luo H, Ge C, Zhang G, Baker RC, Stevenson A, Wiedmann M (2019) Assessment and comparison of molecular subtyping and characterization methods for *Salmonella*. Front Microbiol 10:1591. 10.3389/fmicb.2019.01591 31354679 10.3389/fmicb.2019.01591 PMC6639432

[CR56] Tawakol MM, Nabil NM, Samir A et al (2024) Role of migratory birds as a risk factor for the transmission of multidrug resistant *Salmonella enterica* and *Escherichia coli* to broiler poultry farms and its surrounding environment. BMC Res Notes 17(1):314. 10.1186/s13104-024-06958-7 39420393 10.1186/s13104-024-06958-7 PMC11487717

[CR57] Tawfik RG, Gawish MF, Abotaleb MM, Nada HS, Morsy K, Abumandour MMA, Torky H (2022) Genetic relationship between *Salmonella* isolates recovered from calves and broiler chickens in Kafr El-Sheikh City using ERIC PCR. Animals 12(23):3428. 10.3390/ani12233428 36496950 10.3390/ani12233428 PMC9739306

[CR58] Wang Z, Jiang Y, Xu H, Jiao X, Wang J, Li Q (2023) Poultry production as the main reservoir of ciprofloxacin- and tigecycline-resistant extended-spectrum β-lactamase (ESBL)-producing *Salmonella enterica* serovar Kentucky ST198.2-2 causing human infections in China. Appl Environ Microbiol 89(9):e0094423. 10.1128/aem.00944-23 37610223 10.1128/aem.00944-23 PMC10537671

[CR59] Widodo A, Khairullah AR, Effendi MH, Moses IB, Agustin ALD (2024) Extended-spectrum β-lactamase-producing *Escherichia coli* from poultry: a review. Vet World 17(9):2017–2027. 10.14202/vetworld.2024.2017-2027 39507773 10.14202/vetworld.2024.2017-2027 PMC11536724

[CR60] Williams T, Athrey G (2020) Cloacal swabs are unreliable sources for estimating lower gastrointestinal tract microbiota membership and structure in broiler chickens. Microorganisms 8(5):718. 10.3390/microorganisms8050718 32408567 10.3390/microorganisms8050718 PMC7285018

[CR61] Youssef RA, Abbas AM, El-Shehawi AM, Mabrouk MI, Aboshanab KM (2021) Serotyping and antimicrobial resistance profile of enteric nontyphoidal *Salmonella* recovered from febrile neutropenic patients and poultry in Egypt. Antibiotics 10(5):493. 10.3390/antibiotics10050493 33925773 10.3390/antibiotics10050493 PMC8147078

[CR62] Zhao X, Ju Z, Wang G, Yang J, Wang F, Tang H, Sun S (2021) Prevalence and antimicrobial resistance of *Salmonella* isolated from dead-in-shell chicken embryos in Shandong, China. Front Vet Sci 8:581946. 10.3389/fvets.2021.581946 33796577 10.3389/fvets.2021.581946 PMC8007780

