# Supplementary material for: Molecular characterization of Salmonella isolates from poultry in Egypt: Detection of virulence, ESBLs genes and antimicrobial resistance profiling
Source: Vet Res Commun. 2025 Nov 18;50(1):44. doi: 10.1007/s11259-025-10936-0 (PMC12627184; doi:10.1007/s11259-025-10936-0)
Supplement: Supplementary file 1 — Supplementary Material 1 (DOCX 444 KB) [file 11259_2025_10936_MOESM1_ESM.docx]

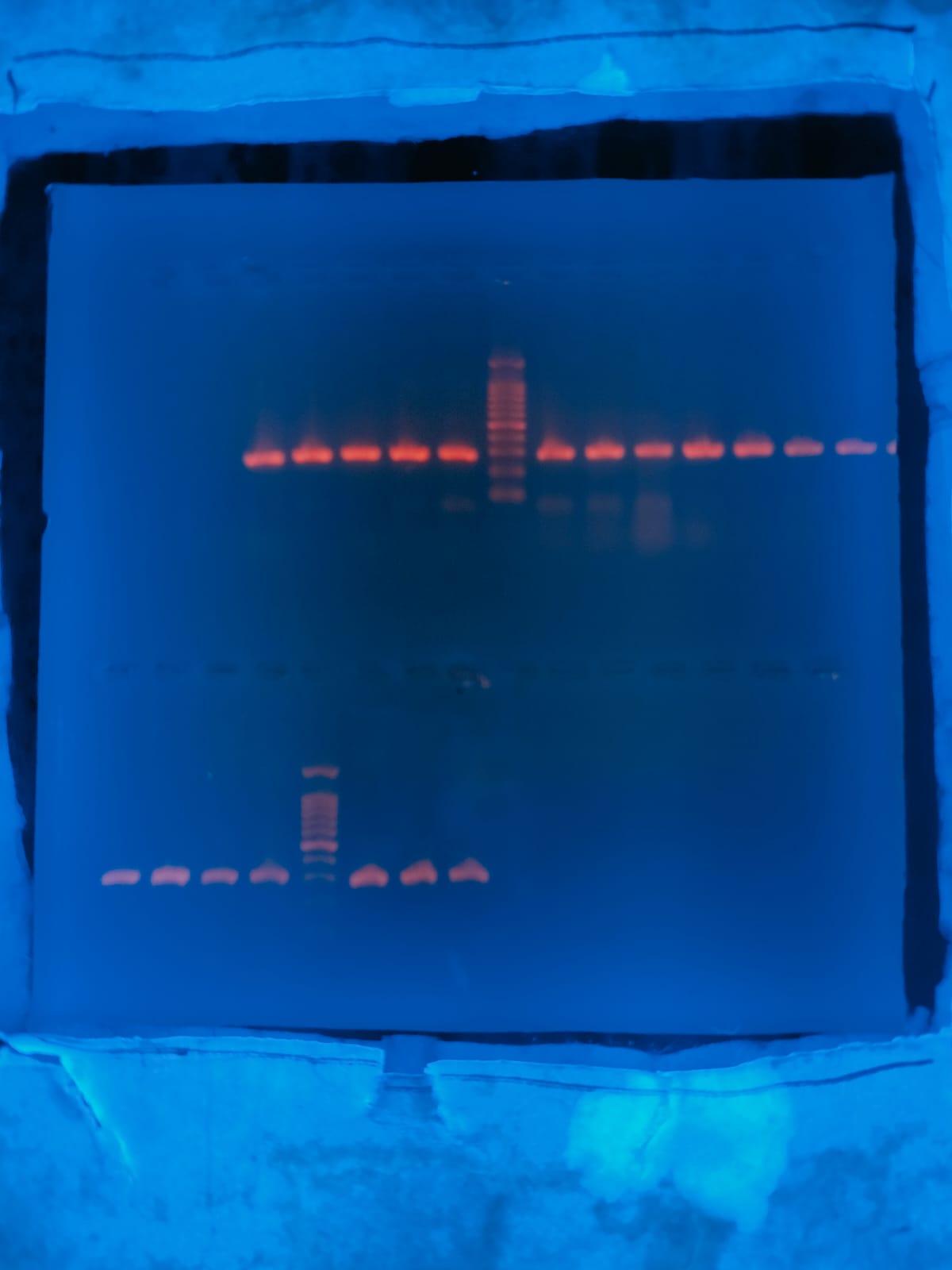


PCR amplification of the *inv*A gene at 284 bp


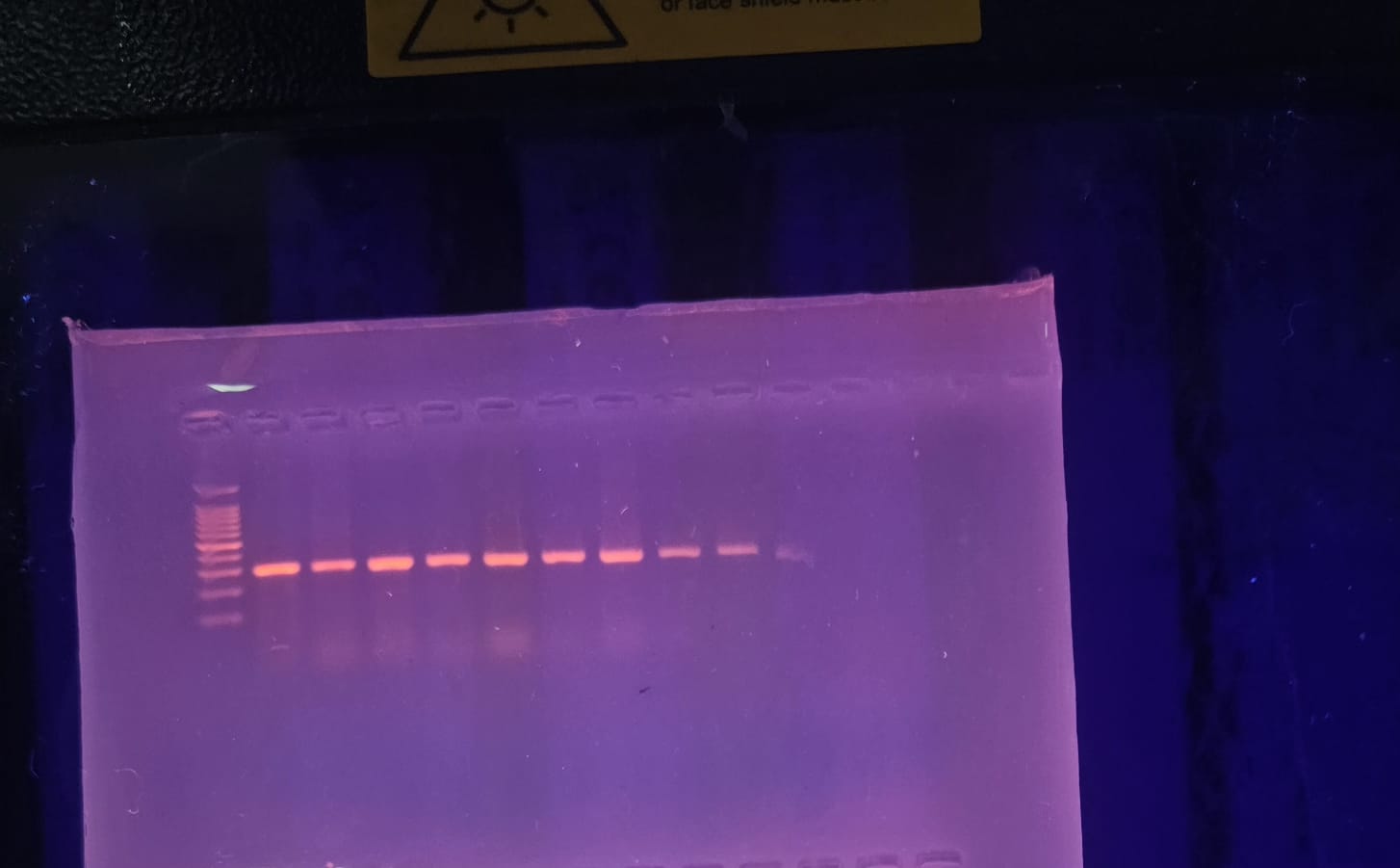


PCR amplification of *hil*A gene at 269 bp


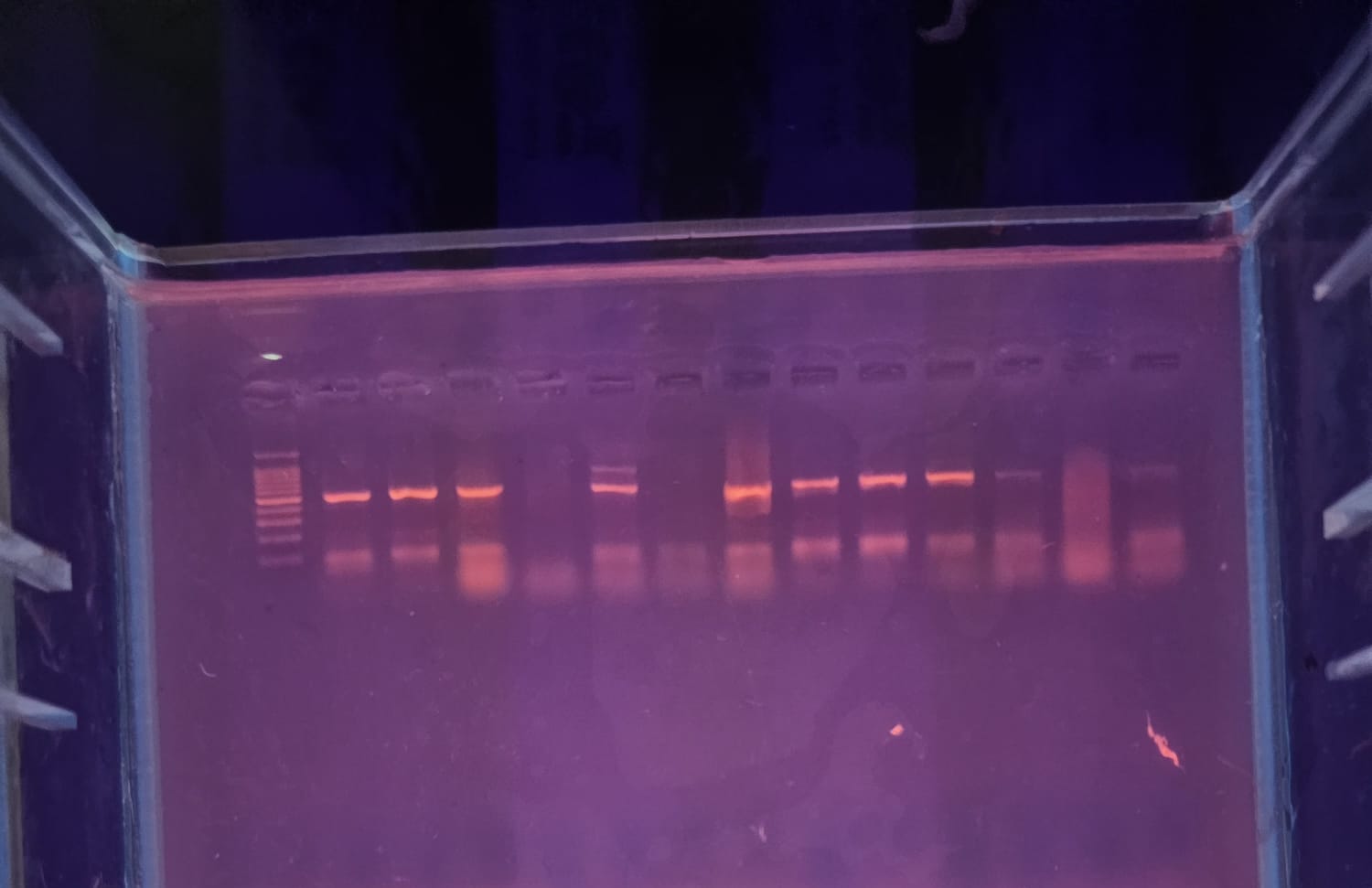


PCR amplification of *stn* gene at 480 bp


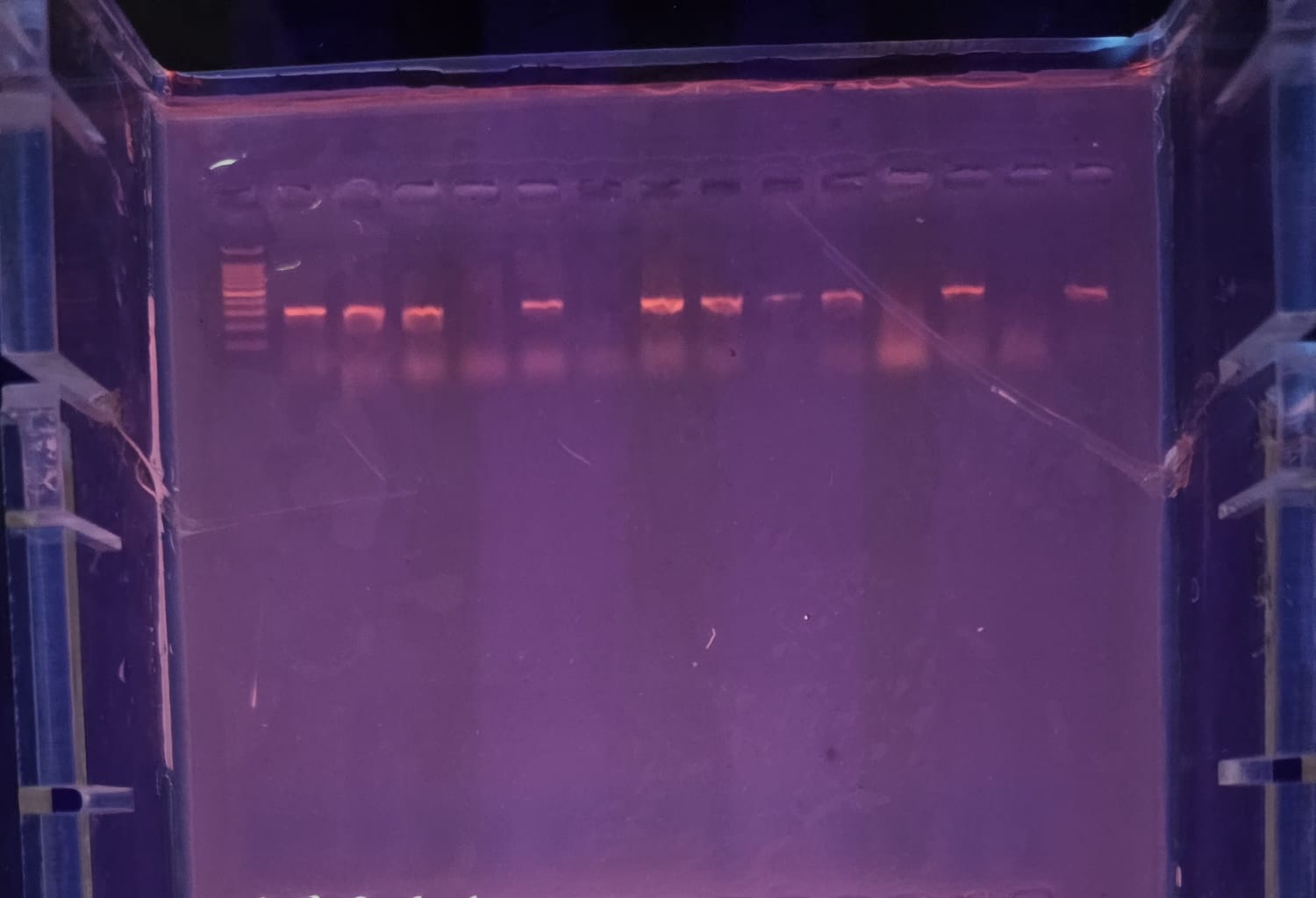


PCR amplification of *spi*C gene at 309 bp


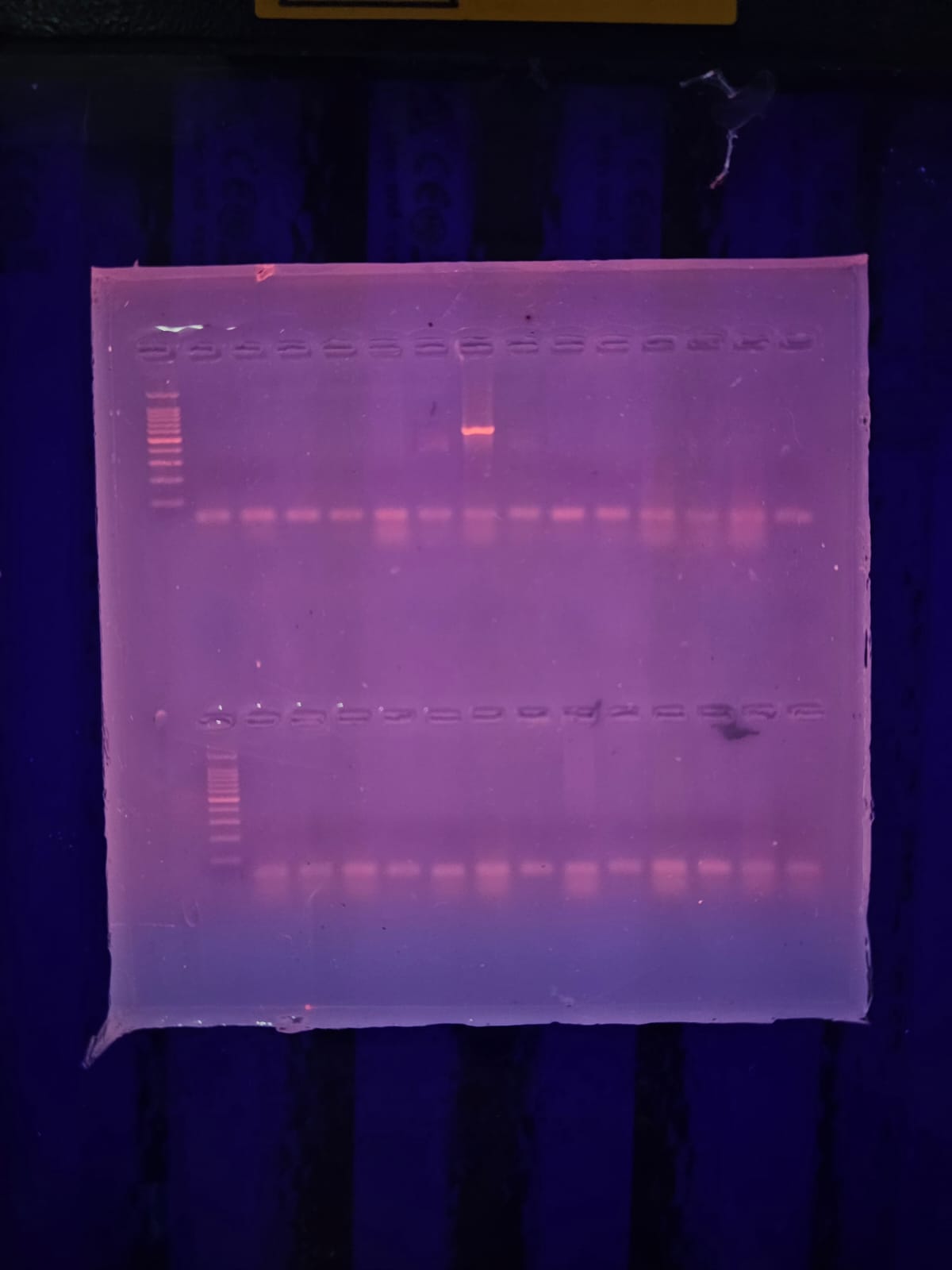


PCR amplification of *spv*C gene at 571 bp.


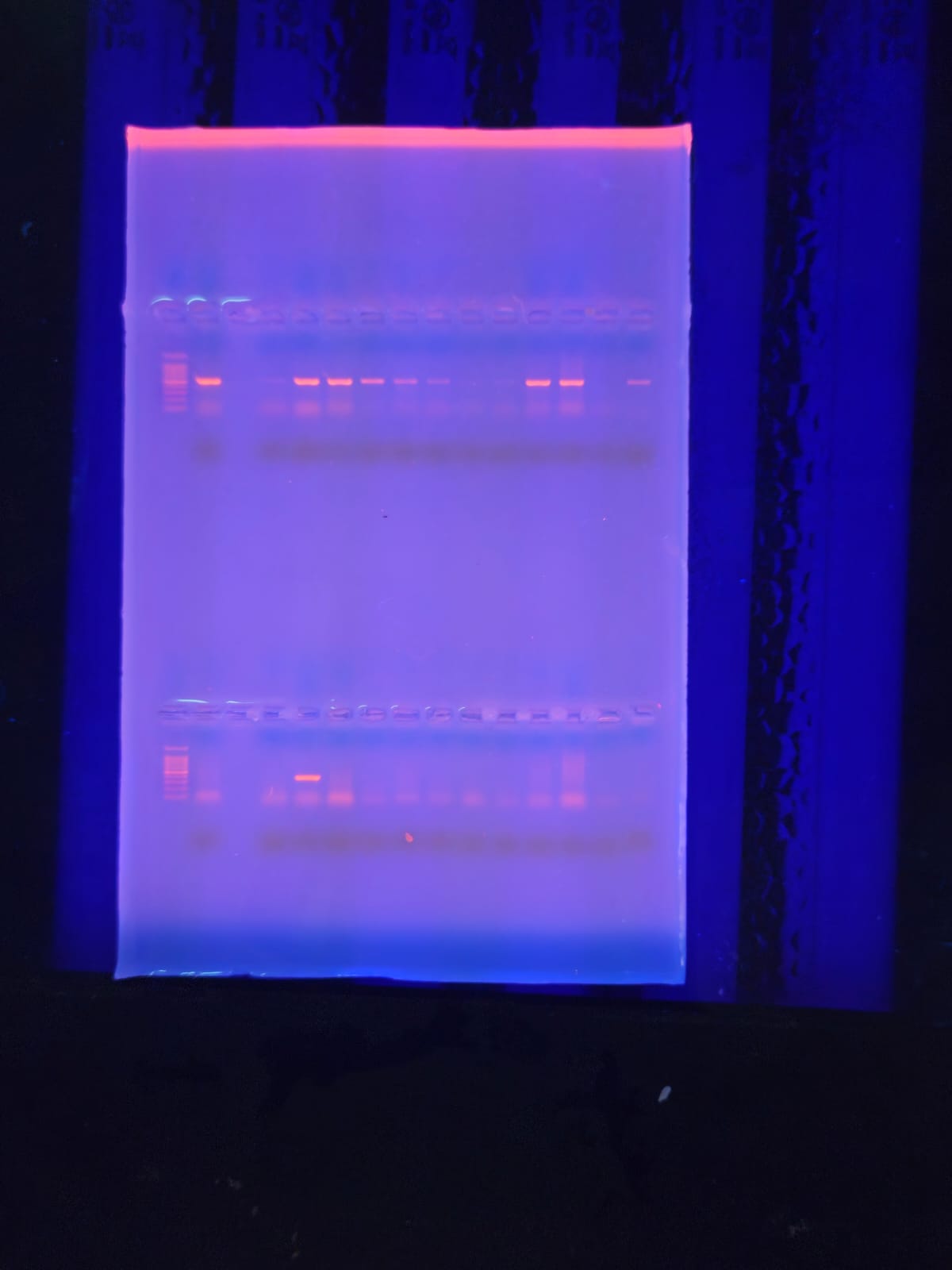


PCR amplification of (A): *bla*_TEM_ gene at 516 bp, (B): *bla*_SHV_ gene at 392 bp.


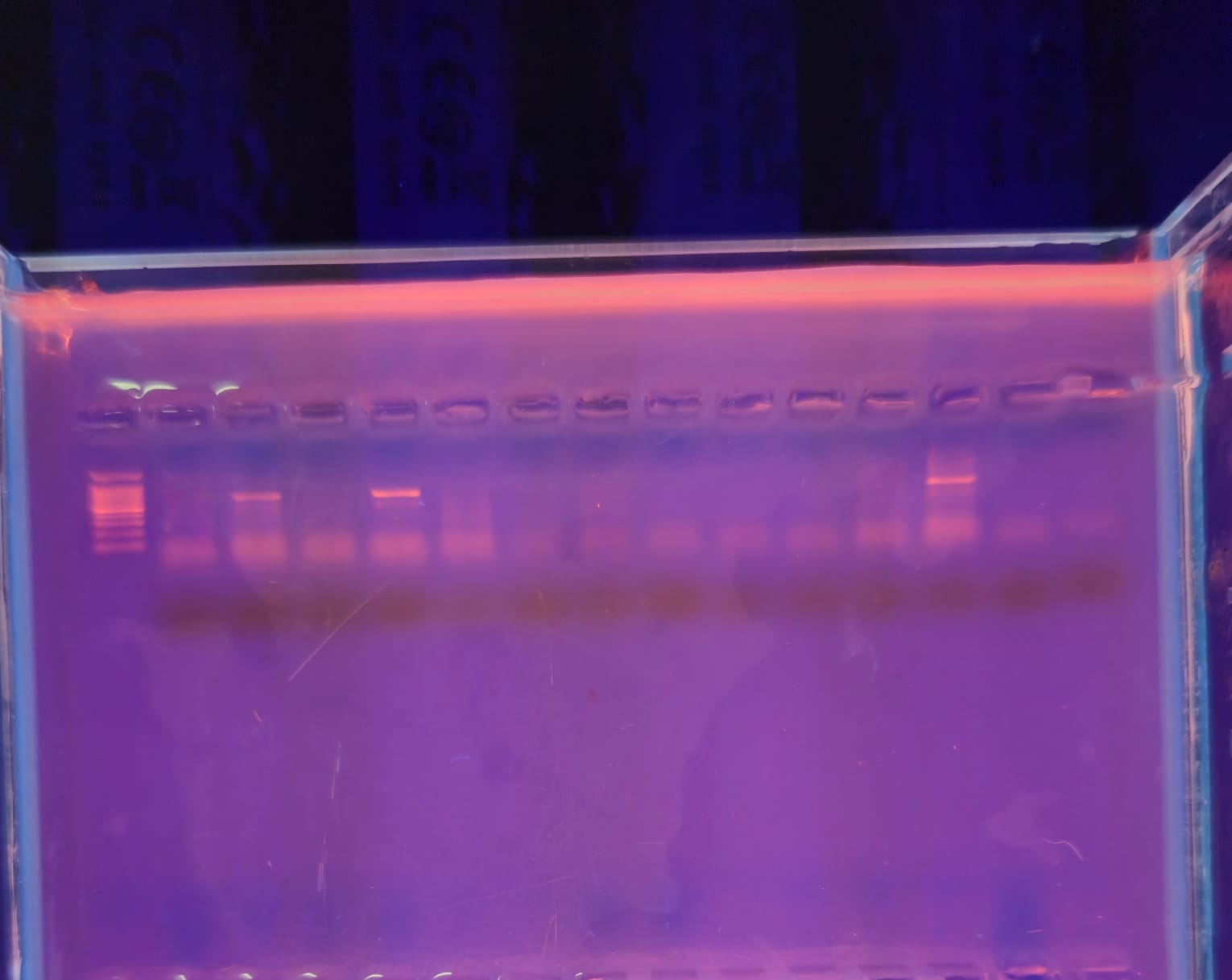


PCR amplification of *bla_CTX-M_* gene at 593 bp.
